# Photoinduced edge-specific nanoparticle decoration of two-dimensional tungsten diselenide nanoribbons

**DOI:** 10.1038/s42004-023-00975-6

**Published:** 2023-08-14

**Authors:** Gennadiy Murastov, Muhammad Awais Aslam, Tuan-Hoang Tran, Alice Lassnig, Kenji Watanabe, Takashi Taniguchi, Stefan Wurster, Manfred Nachtnebel, Christian Teichert, Evgeniya Sheremet, Raul D. Rodriguez, Aleksandar Matkovic

**Affiliations:** 1 Chair of Physics, Department Physics, Mechanics and Electrical Engineering, Montanuniversität Leoben, Franz Josef Strasse 18, 8700 Leoben, Austria; 2 Tomsk Polytechnic University, Lenina Avenue 30, 634034 Tomsk, Russia; 3 Erich Schmid Institute of Materials Science, Austrian Academy of Sciences, Jahnstrasse 12, 8700 Leoben, Austria; 4 Research Center for Functional Materials, National Institute for Materials Science, 1-1 Namiki, Tsukuba, 305-0044 Japan; 5 International Center for Materials Nanoarchitectonics, National Institute for Materials Science, 1-1 Namiki, Tsukuba, 305-0044 Japan; 6 Graz Centre for Electron Microscopy (ZFE), Steyrergasse 17, 8010 Graz, Austria

**Keywords:** Nanoparticles, Two-dimensional materials, Surface assembly, Photocatalysis, Surface patterning

## Abstract

Metallic nanoparticles are widely explored for boosting light-matter coupling, optoelectronic response, and improving photocatalytic performance of two-dimensional (2D) materials. However, the target area is restricted to either top or bottom of the 2D flakes. Here, we introduce an approach for edge-specific nanoparticle decoration via light-assisted reduction of silver ions and merging of silver seeds. We observe arrays of the self-limited in size silver nanoparticles along tungsten diselenide WSe_2_ nanoribbon edges. The density of nanoparticles is tunable by adjusting the laser fluence. Scanning electron microscopy, atomic force microscopy, and Raman spectroscopy are used to investigate the size, distribution, and photo-response of the deposited plasmonic nanoparticles on the quasi-one-dimensional nanoribbons. We report an on-surface synthesis path for creating mixed-dimensional heterostructures and heterojunctions with potential applications in opto-electronics, plasmonics, and catalysis, offering improved light matter coupling, optoelectronics response, and photocatalytic performance of 2D materials.

## Abstract (web summary)

Metallic nanoparticles are widely explored for boosting light-matter coupling of 2D materials, however, the target area for nanoparticle deposition is typically restricted to either the top or the bottom of 2D flakes. Here, the authors show tunable, edge-specific nanoparticle decoration using a laser, achieving arrays of silver nanoparticles that are self-limited in size along tungsten diselenide nanoribbon edges.

## Introduction

Metallic nanoparticles (NPs) are widely used in photocatalysis, surface-enhanced Raman spectroscopy (SERS), and plasmonics, due to their capability to strongly couple the incoming light to a plasmonic response^1–5^. To harness light-matter interaction in 2D material transition metal dichalcogenides (TMDCs)^6,7^ they’re combined with NPs via different approaches including drop-casting^8^, dip-coating^9,10^, atomic layer deposition^11,12^, self-assembled layer^13,14^, and spontaneous redox reaction^15^. Yielding with mixed-dimensional heterostructures^16^, hybrid materials^17^, and heterojunctions^18^ that have been effectively employed for various applications ranging from hydrogen evolution reaction (HER) to light-emitting diodes and sensors^19–25^. For example, molybdenum disulfide (MoS_2_) with deposited Au@Ag co-shell nanorattles attenuated overpotential and reduced Tafel slope for the HER due to plasmonic hot electron photocatalysis tuned by laser power and wavelength^26^.

Commonly in these hybrid structures, both the basal plane and the edges of 2D materials are covered with NPs to optimize them for SERS and optoelectronic devices with large active surface area^27–31^. This has been demonstrated by Rahaman et al., where they show localized surface plasmon resonance assisted by hot electron transfer, resulting in anomalous Fröhlich interaction^32^. However, there are several drawbacks of the aforementioned methods such as non-selective deposition, basal plane crystallinity disruption of 2D materials and their straining.

2D material-based nanoribbons (NRs) provide a promising platform to overcome these issues, as these systems offer a high edge-to-surface ratio. Since the edges predominantly serve as nucleation sites, the basal plane coverage is minimized. NRs have been employed for targeted drug delivery, molecular and gas sensing, filtering, (photo-)catalytic reaction, electronics, and optics^24,33–38^.

Recently, spatially controlled deposition of metallic NPs and “nanoflowers” was demonstrated for transparent and non-transparent substrates utilizing the laser-induced photo-decomposition of [{Au_10_Ag_12_(C_2_Ph)_20_}Au_3_(PPh_2_(C_6_H_4_)_3_PPh_2_)_3_] [PF_6_]_5_ complexes in different solutions^39,40^. Also, the high-precision patterning ability of the spot scanning system was utilized to selectively deposit NPs on the topmost surface of MoS_2_ 2D flakes via light-induced photoreduction of silver nitrate AgNO_3_ solution^41^. The NPs were found more likely to be attached to the chemically active sides, i.e., intrinsic and laser-induced defects regions^41^. Therefore, the edges of the flakes or the 2D material-based NRs are expected to serve as primary nucleation sites for silver seeds. This was observed in our previous study for graphene NRs decorated by metallic NPs via the photo-activated reduction of Ag ions at the edges via electron transfer from graphene^42^.

In this work, we perform silver nanoparticle (AgNP) edge-specific decoration of WSe_2_ flakes and their NRs. We demonstrate the tunable particle density along the NR edges in-line with the laser fluence. Protected with an organic mask—a by‑product of the NR fabrication method—the basal plane of NR remains encapsulated while the NR edges are simultaneously decorated with metallic NPs. Obtained hybrid systems have shown the enhancement in Raman signal and photocatalytic activity by conversion of 4-nitrobenzenthiol (4-NBT) to p, p′-dimercaptoazobenzene (DMAB).

## Results and discussion

### Edge-specific decoration of 2D NR networks

The NR networks of WSe_2_ were prepared by a method previously established by Aslam et al. in ref. ^42^. It utilizes needle-like nanostructures of para-hexaphenyl C_36_H_26_ (6P, p-6P) grown on top of the 2D flakes and acts as a mask for the following oxygen plasma treatment.

Here, we employ a single-step treatment to simultaneously reduce, merge and deposit AgNPs in the silver ionic solution via direct light irradiation of immersed NR samples. Moreover, we vary the power of a continuous-wave 637 nm laser from 200 μW to 35 mW and adjust the scanning speed from 5 to 50 μm s^-1^ to tune the total applied laser fluence in the range of 1 to 300 μJ μm^-2^ (detailed information is given in the Supplementary Note 1).

**Fig. 1:**
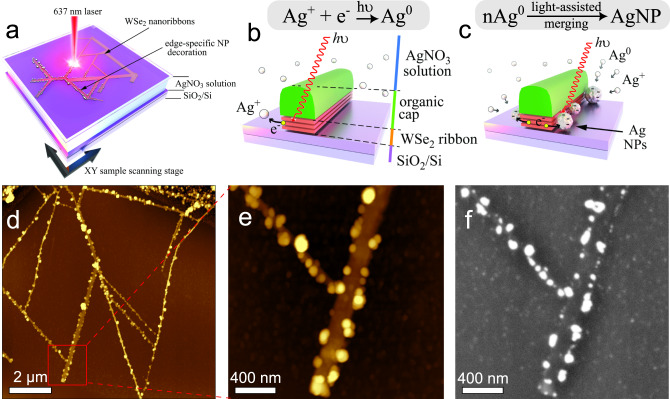
Edge-specific WSe_2_ nanoribbons decorated with silver nanoparticles. **a** The sketch of the laser-scanning NP’s deposition process in the silver ionic solution. **b** The main mechanism of ‘seeds’ formation via silver ions reduction under the strong laser fluence. **c** The light-assisted NP merging on the edge of NR. **d** The overview (AFM topography, z scale is 60 nm) of the WSe_2_ NR network decorated with AgNP. **e**, **f** A zoom-in region marked by the red square in **d** presenting the surface topography (AFM, z scale is 28 nm) and scanning electron microscopy (SEM) images of the WSe_2_ NR network decorated with AgNPs.

Aiming to understand the mechanism behind the NP formation and to emphasize the role of the 2D material edges, we focus on the edge-specific decoration of the photoactive quasi-1D WSe_2_ NRs. The 6P organic masks remained on top of the ribbon’s networks to prevent tungsten oxide formation during laser exposure^43,44^. The organic ‘caps’ also help to enhance the edge-selectivity of silver NPs deposition. The scheme of the deposition process and the double-side arrays of AgNPs obtained by atomic force microscopy (AFM) and scanning electron microscopy (SEM) are presented in Fig. 1.

Direct laser illumination of the AgNO_3_ solution does not create NPs while in the presence of WSe_2_ the light triggers the reduction of a silver ion to the neutral state due to the release of photo-excited electrons from NRs. Ag^0^ seed acts as a source for further NPs growth. The NR cross-section view is schematically illustrated in Figs. 1b, c with a single-step reduction of silver ions and NP assembly, respectively. As the electrons originate mostly from the edge defects of the 2D TMDC NRs, the seeds are anchored to them, and the decoration with AgNPs remains exclusive to the edges. Also, the organic ‘caps’ on top of NRs serve as a shield and a guide for the NPs deposition. An overview of the obtained WSe_2_ NR network decorated with NPs anchored to the ribbon edges is presented in Fig. 1d.

Figures 1e, f present in higher magnification images of WSe_2_ NR junction with AgNPs. Most of the AgNPs stick to the edges. In the case of the TMDC NR networks, and for the properly tuned growth parameters, non‑edge specific nucleation or shifting of the NPs during the rinsing steps was found to occur for less than 1% of all detected NPs within the illuminated area. At the same time, the basal plane of the NRs is protected by the organic layer, which prevents any NP deposition on the NR top surface.

**Fig. 2:**
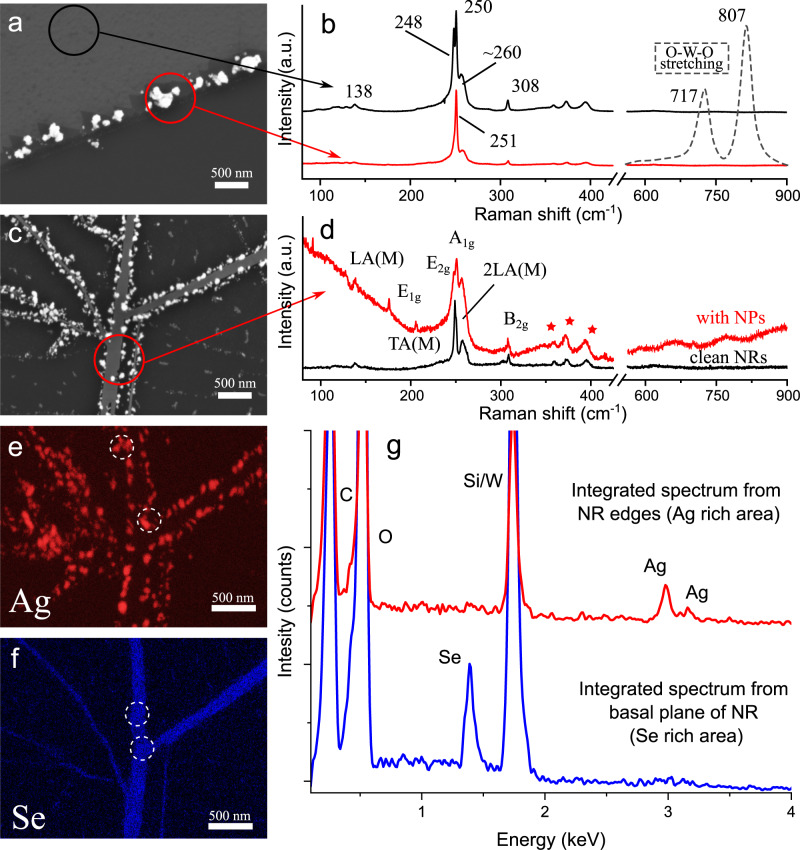
SEM and Raman spectroscopy on the WSe_2_ flake and nanoribbon network with edge-specific decoration by silver nanoparticles. **a** WSe_2_ flake’s edge-specific assembly of the AgNPs with a laser fluence of ~1.3 μJ μm^-2^. **b** Comparison of Raman spectra between the intact area and NP decorated edge. The SERS signal on Ag(25 nm)-WO_3_ system (dashed line) was adapted with permission from ref. ^49^. © 2014 Springer Nature. **c** WSe_2_ NR’s edge-specific AgNPs decoration with a laser fluence of ~100 μJ μm^-2^. This region was laser scanned twice to obtain more NPs. **d** The Raman spectra with and without NP deposition. The Raman peaks marked with the red stars are attributed to the second-order features excited with the double and the triple resonance mechanisms due to plasmon resonance^46^. EDX elemental maps performed for silver **e** and selenium **f** at the same area as on panel **c**. **g** Corresponding energy dispersive X-ray spectra to the NR edges (Ag rich area) and NR basal plane (Se rich area).

The NRs retain most of the structural and electronic properties of the original 2D TMDC flakes^42^. Therefore, the same edge‑specific decoration process was also tested on the non‑patterned WSe_2_ flakes without an organic mask. The edges of the flakes, with respect to the basal plane of the 2D sheet, inevitably introduce intrinsic inhomogeneity and defect complexes. Therefore, the edge‑specific decoration process also occurs for the flakes, as demonstrated for the edge of the mechanically cleaved WSe_2_ layers (Fig. 2a). Corresponding Raman spectra obtained from the bare flake and the NPs on the edge are presented in Fig. 2b.

Usually, the Raman spectrum of WSe_2_ is described by several peaks originating from the interlayer breathing mode A_1g_, the in-plane displacement E_1g_ of the chalcogen atoms, the E_2g_^2^ shear mode corresponding to the vibration of two rigid layers against each other^47^. The overtone of the LA phonon branch at the M point of the Brillouin zone can be observed in Raman. It is commonly noted as 2LA(M) alongside the prominent B_2g_^1^ peak and is associated with an A-symmetry first-order mode corresponding to an interlayer vibration^18,45–48^. In Fig. 2b, the presence of the A_1g_ (250 cm^-1^), E_2g_^2^ (248 cm^-1^), B_2g_^1^ (308 cm^-1^), 2LA(M) (~260 cm^-1^) modes together is attributed to the thickness of 3 trilayer (3TL) WSe_2_^45^.

Near the NP decorated edges, A_1g_ and E_2g_^2^ were found to form a single A + E mode at ~251 cm^-1^ associated with the out-of-plane vibration in WSe_2_. No laser-induced tungsten (tri-)oxides WO_3-x_ formation^49^ were found supported with almost zero-line Raman in Fig. 2b. The dashed line represents O-W-O stretching modes at 717 cm^-1^ and 807 cm^-1^ for the hybrid system of Ag(25 nm)-WO_3_^49^.

Figure 2c demonstrates one of the densest NP-decorated networks obtained in our experiments. Corresponding Raman spectra of AgNP decorated WSe_2_ NRs and untreated NRs are presented in Fig. 2d. It contains the unique features coming from inactive resonant phonons such as E_1g_ (~175 cm^-1^), TA(M) (~205 cm^-1^) which could be attributed to the z¯(xx)z polarized configuration in double and multilayer system of WSe_2_^45,48^. Further, decorated NRs exhibit the second‑order features of WSe_2_ Raman modes due to double and triple plasmon resonance mechanism^46^ and are obtained as enhanced peaks at ~358 cm^-1^, ~372 cm^-1^, and ~394 cm^-1^. Lastly, compared to the untreated WSe_2_ NRs, the decorated ones bring a strong luminescence background in the Raman signal assigned to the possibility of the heterojunction formation^18^.

The same WSe_2_ NR network as for Fig. 2c was analyzed by energy dispersive X-ray (EDX) spectrometry. The presence of silver is confirmed by tracking the Ag L peak during the map measurements. To obtain a better signal-to-noise ratio, spectral map points from the areas labeled with dashed circles in Fig. 2e were merged and are presented as an integrated spectrum in Fig. 2g. The same is done for Se peak from the basal plane of the NR given in Fig. 2f. Applying the edge‑selective NP decoration process to the NR networks benefits from their enhanced edge‑to‑surface ratio^42^ and provides a possible pathway to incorporate these hybrid nanostructures in sensing and catalytic applications^19,36,38^.

### AgNP distribution

**Fig. 3:**
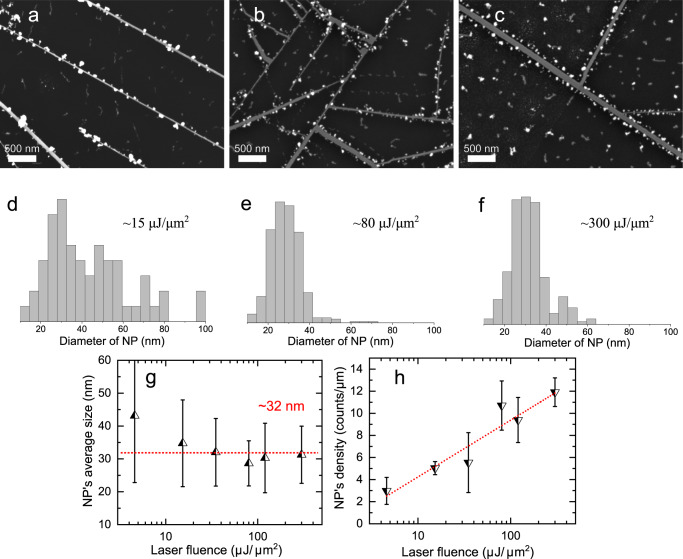
Multilayer WSe_2_ nanoribbon networks irradiated with different laser fluences. SEM images of AgNPs deposited with a relatively low laser fluence of **a** ~15 μJ μm^-2^, **b** ~80 μJ μm^-2^ and **c** ~300 μJ μm^-2^. **d**, **e**, and **f** NPs size distribution (diameter of AgNP) calculated from **a**, **b**, and **c**, respectively. The counts are normalized to one to have a uniform scale. **g** The average deviation of AgNP size along the NR edges. **h** The evaluated linear NP density depends on the applied laser fluence. Irradiation with a laser fluence of more than 400 µJ μm^-2^ leads to NP agglomeration. The error bars in **g**, **h** present a standard deviation.

To exploit the tunability of the light-assisted deposition method over the AgNP’s distribution in the patterned areas, we performed a series of experiments with different laser power, time, and scanning speeds. The results presented in Fig. 3 are given as a function of laser fluence, while all varying parameters are included in Supplementary Table 1. A fairly good stability and reproducibility in size were achieved, especially considering that the proposed edge-specific laser-treatment method is a solution-based process (Fig. 3d–f). Also, NPs growth was found to be very similar on both predominantly zigzag and armchair edge types. This can be seen at ~90° joints between the NRs from Fig. 3b, c (more details in Supplementary Fig. 1). There are still a few NPs that could be found not anchored to the edges due to thermal fluctuations and diffusion in water.

The size distribution of the deposited AgNPs slightly deviates between the samples (see the error bars in Fig. 3g). Surprisingly, the average NP diameter with a mean value of ~32 nm remains the same for the laser fluence up to 300 μJ μm^-2^. This is attributed to the limited number of seeds available in a short reaction time. Essentially, the self‑stabilizing particle size would support our hypothesis that the creation of new nucleation spots prevails as a dominant mechanism over the merging process of the NPs. This is further supported by the fact that NP merging is rather slow^50,51^.

Also, we found the correlation between the laser fluence and the linear NP density along the NR edges. We did not observe a lot of agglomerates and large particles among the deposited NPs, instead, the NPs nucleation linear density varied from ~2 to ~12 NPs per μm of the NR length as laser fluence increased (Fig. 3h). The results imply that this is a self-saturated edge-driven process, i.e., the reduced Ag^+^ competes for the edge’s nucleation spot leading to the dense deposition instead of the larger particle formation. Contrary to this, in a colloidal solution the triangle- and the rod-shaped NPs within hundreds of nm in size could be obtained via the plasmon oscillation enhancement merger in a few hours of the continuous light-annealing^50–52^.

It should be mentioned that no edge decoration of WSe_2_ NRs capped with 6 P is observed with the laser fluence less than ~5 μJ μm^-2^. Compared to the WSe_2_ flake, 5 times higher fluence is needed to induce edge-specific AgNPs formation on NRs.

### The sources of photo‑excited electrons and the influence of the organic layer on the NP growth

As we highlighted before, the WSe_2_ edges are a key factor to perform the AgNO_3_ reduction and to anchor the NPs to the edges of the NR networks. In the performed NP growth experiments, the 6P organic cap remained partly on the NR’s basal plane. Further, 6P is known as a photoluminescent material^53^. Therefore, 6P caps should also be able to initiate the Ag seed formation assisted by strong coherent light.

**Fig. 4:**
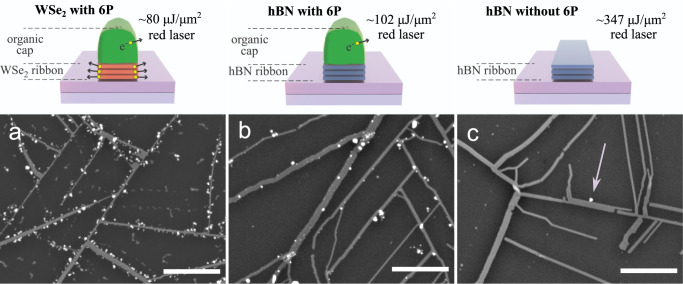
SEM images of the WSe_2_ nanoribbon and hBN nanoribbon networks with and without para-hexaphenyl organic mask. **a** AgNP’s double-edge array on the WSe_2_ NR network with 6 P on top. **b** hBN NR with 6P on top of the network immersed in the AgNO_3_ solution and irradiated with a red laser (637 nm). **c** No AgNPs deposited on the cleaned hBN NR network. The scale bar is 1 μm.

To investigate the impact of 6P on the proposed laser‑assisted NP growth, we have performed a negative experiment on hexagonal boron nitride NRs with and without 6P caps (Fig. 4). Since hBN is an insulator (~6 eV bandgap)^54^, we can assume that photo‑excited electrons cannot be generated with our ~1.9 eV laser. However, essentially identical 6P organic nanostructures can be grown on hBN^55^ and hBN NR networks can be fabricated using the same method as was employed for WSe_2_ NRs^42^. Furthermore, hBN is more thermally stable and can withstand the prolonged vacuum annealing needed to desorb 6P^56^. This allowed us to test the NP decoration on clean hBN nanoribbons, as a negative experiment. Therefore, we could test the influence of the 6P caps in NP seeding.

Both WSe_2_ and 6P can actively support the growth of NPs on the edges by donating the excited electrons and promoting the nucleation centers, yielding the dense AgNPs NR edge decoration at ~80 μJ μm^-2^ (Fig. 4a). Also, the hBN NRs covered with 6P enable the AgNPs decoration with the laser fluence of ~102 μJ μm^-2^ (Fig. 4b). However, at higher fluence (above 400 µJ μm^-2^) 6P decomposition starts to occur providing an additional electron source for the reduction process. This yields also non-edge-specific deposition on the ribbon’s basal plane as well as on the bare substrate near the ribbons.

The deposition process on hBN capped with 6P is ~5 times less efficient in comparison to WSe_2_ (Fig. 4a, b) as the process is mostly driven by the 6P source. This can be simply attributed to the fact that hBN, unlike WSe_2_, is not promoting the nucleation of AgNPs. Opposite results were obtained for MoS_2_ and WS_2_ NRs. In these cases, and within the explored parameter space, the sulfide-based 2D materials were easily oxidized forming the Ag_2_MoO_4_ dendrites and WO_*x*_, Ag_2_S, structure as shown in Supplementary Figs. 2–3.

Finally, as a confirmation that the NP growth process is also partly driven by the photo‑excited electrons from the organics, the decoration process was repeated on hBN NRs without 6P caps (Fig. 4c). Almost zero AgNPs were observed on the cleaned hBN NRs, even with a higher laser fluence ~347 μJ μm^-2^ applied. Interestingly, the same finding was obtained on PtSe_2_ NRs likely due to lower light-matter interaction. Still, randomly sedimented NPs were occasionally observed, but no systematic deposition, as pointed in Fig. 4c, what can be attributed to a liquid-based process and the presence of natural contaminants in the solution.

### Light-mediated catalysis on AgNPs decorated edges

As a potential system for HER, the conversion of 4-NBT to 4-ABT and p, p′-dimercaptoazobenzene on plasmonic NPs are highly used in big pharma industries^57–59^. Here, we demonstrate the possibilities of our AgNP-decorated NRs as a platform for the photocatalytic conversion of 4-NBT molecules to DMAB.

**Fig. 5:**
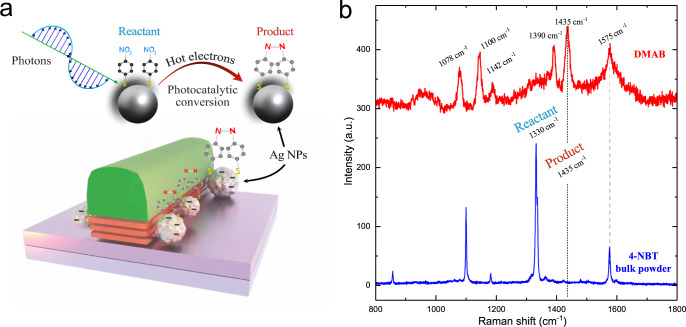
Demonstration of photoactive silver nanoparticles behavior via photocatalysis of 4-nitrobenzenthiol probe molecules into p, p′-dimercaptoazobenzene with the green Raman laser (532 nm). **a** The dimerization reaction of 4-NBT on metallic NPs via ‘hot’ electron doping. **b** Raman spectra of 4-NBT powder and 4-NBT laser-induced conversion to DMAB in the presence of AgNP at the NR edge.

Raman spectroscopy allows us to track chemical composition changes in real-time in a non-destructive manner. No molecular degradation, peak shifting, or quenching of the Raman peaks from the 4-NBT powder was observed with direct laser exposure up to 100 mW for a 532 nm wavelength laser irradiation. For the catalysis experiments, we used freshly prepared AgNPs to minimize the metal oxide formation in ambient conditions. Figure 5a illustrates the plasmon-assisted surface catalytic reaction to perform the dimerization of 4-NBT to DMAB. By monitoring the main peak located at ~1330 cm^−1^ assigned to the symmetric stretching vibration of the nitro group ν(NO_2_) in 4-NBT, we could track the degree of conversion. The other distinct peaks in Fig. 5b are attributed to the C–C ring stretching (~1575 cm^−1^) and C−S vibration (~1100 cm^−1^)^60,61^.

After laser irradiation in the presence of AgNPs, all Raman peaks, except ~1575 cm^−1^ one, change drastically. The new combined peak of νCC + δCH ring vibration appeared at ~1435 cm^−1^, evidencing the successful photocatalytically driven DMAB formation as shown in Fig. 5b.

These experiments confirm that the deposited AgNPs via the proposed edge-specific method show high photocatalytic activity. Almost all of 4-NBT was transformed to the DMAB after a few seconds under 0.1 mW@532 nm Raman laser as no ν(NO_2_) peak was visible anymore in the Raman spectra (Fig. 5b, black curve). The NP-decorated NRs perform similarly to conventional AgNP-based SERS-substrates^62,63^.

### Conclusion

We proposed a solution‑based method for edge‑specific laser-assisted NP formation. This method is demonstrated using 2D WSe_2_ flakes and their NRs. Photons excite the electrons at the TMDC edges immersed in the AgNO_3_ solution, reducing the silver ions and leading to the formation of AgNP seeds that grow and remain anchored to the edges. This method is pushed to the limit by employing nanopatterned networks of WSe_2_ NRs that enhance the edge‑to‑surface ratio by orders of magnitude compared to the pristine 2D flakes. A constant laser fluence is needed to merge the seeds into NPs. This results in double linear arrays of NPs along the NRs with an average diameter of ~32 nm, which is attributed to short reaction time and a limited number of seeds. Remaining the average size of NP fixed, the linear NPs density along the NR edge can be controlled by tuning the laser fluence. Freshly deposited AgNPs result in an increase in the Raman signal of WSe_2_ NRs and an enhancement of the resonant modes. Further, the high photocatalytic activity of the AgNPs/NRs system was demonstrated by the full conversion of 4-NBT to DMAB.

The edge-selective decoration of metallic NPs is an approach for building mixed-dimensional heterostructures and heterojunctions with high lateral precision and synergy between optoelectronic properties of 2D materials and the strong light coupling of plasmonic NPs. We expect that this will yield much better electronic properties of the WSe_2_ NRs than of the basal plane decorated thin films. Decoration of the basal plane can lead to damage of the 2D semiconductor crystal structure and therefore an increased scattering rate of the free carriers. Consequently, the electronic properties of the 2D semiconductor could be significantly degraded making potential electronic excitation or readout impossible.

This laser-assisted decoration method can be extended and used in tandem with other strategies and other 2D materials to develop, pattern, enhance and investigate the complex micro- and nano-properties of materials and systems. Our proposed edge‑specific and laser‑assisted NP decoration method of 2D materials opens a new pathway to develop ultra-sensitive devices based on the hybrid heterostructures and heterojunctions between 2D materials, their reactive edges, and metallic NPs, with potential impact mainly on optoelectronic, catalytic, and plasmonic systems.

## Methods

### NR network fabrication

NR network fabrication was described in ref. ^42^. Briefly, hot wall epitaxy was used to deposit the organic mask containing 6P (p-6P or C_36_H_26_ consists of six phenyl rings connected by single sp² carbon–carbon bonds forming a rod-like conjugated molecule) on top of 2D flakes (WSe_2_, MoS_2_, WS_2_, graphene, and hBN) followed by reactive oxygen ion etching. The obtained NR networks were further used for AgNP decoration.

### Solution preparation

The silver nitrate was purchased from Sigma Aldrich (99.9 % chemical grade, powder). 1.7 mg of AgNO_3_ was dissolved in 10 ml of DI water to obtain the 1 mM silver ionic solution used for further NPs deposition.

### Deposition setup

A homemade 3D-printed microscope coupled with a red laser diode (RLT 635-180MGE, with a central peak wavelength of 637 nm, a maximum power of 180 mW) was used for the laser treatment of the sample. The laser power was controlled by Keithley 2400 current source meter. The average spot size given by the 20× objective was estimated to be 5 μm. A 3D printed XY stage^64^ allows the sample scanning with respect to the fixed laser spot position.

### 6P cleaning

The NR networks with organic masks on top were annealed in high vacuum (10^-6^ mbar) with a constant temperature of 200 °C for at least 6 h. The successful removal of 6P was confirmed by Raman spectroscopy and by comparing the AFM images before and after heating.

### Atomic force microscopy (AFM)

The samples were measured using the scanning probe microscope Horiba/AIST-NT Omegascope AFM system in ambient conditions. Topography profiles were obtained in tapping mode with high-frequency probes Nunano Scout (spring constant of 42 N m^-1^, resonant frequency ~ 350 kHz, and tip radius of 5 nm).

### Scanning electron microscopy (SEM)

The scanning electron microscopy micrographs were obtained using a field-emission scanning electron microscope Zeiss LEO 1525 to evaluate the NP size and distribution. The micrographs were recorded with an acceleration voltage of 20 kV and an aperture size of 60 μm in secondary electron detection mode.

### EDX mapping

Zeiss Sigma 300 VP coupled with EDS Detector Oxford SDD 80 was used to obtain the elemental map from the decorated WSe_2_ nanoribbons covered within 5–10 nm carbon coating to minimize surface changing and image drifting during the long-term measurements.

### NP edge-decoration and photocatalysis experiments

5 μl of 1 mM AgNO_3_ were dropped on the samples. The thin glass was used to reduce water evaporation during the laser scanning. The constant spacing of 400 μm between the surface and glass was maintained by polydimethylsiloxane small sheets (Gel-Pak-DLG-X4) attached to the edges of Si substrate. The 3D-printed deposition setup is used for patterning.

For the photocatalytic experiment, the 4-NBT powder was dissolved in DI water mixed with ethanol in a ratio of 50:50 to have a concentration of 0.1 mM. 2D NR networks decorated with AgNPs were immersed into a solution for several hours and rinsed with DI water several times prior to Raman measurements.

### Raman spectroscopy

Raman measurements were performed using a Horiba LabRam HR Evolution confocal Raman spectrometer using 600 lines mm^-1^ and 1800 lines mm^-1^ gratings. A 532 nm laser source was used to excite the samples with an excitation power of 0.1–3.2 mW. The laser spot was focused by a 100×, 0.9 NA objective.

### Supplementary information

Peer Review File

Supplementry Information

## Data Availability

The authors declare that all the data supporting the findings of this study are available within the article or available from the corresponding authors on reasonable request.
