# Supplementary material for: Photoinduced edge-specific nanoparticle decoration of two-dimensional tungsten diselenide nanoribbons
Source: Commun Chem. 2023 Aug 14;6:166. doi: 10.1038/s42004-023-00975-6 (PMC10425467; doi:10.1038/s42004-023-00975-6)
Supplement: Supplementary file 2 — Supplementry Information [file 42004_2023_975_MOESM2_ESM.pdf]

Supplementary Information

Photoinduced edge-specific nanoparticle decoration of two-dimensional tungsten diselenide nanoribbons

Gennadiy Murastov[1*], Muhammad Awais Aslam[1], Tuan-Hoang Tran[2], Alice Lassnig[3], Kenji Watanabe[4], Takashi Taniguchi[5], Stefan Wurster[3], Manfred Nachtnebel[6], Christian Teichert[1], Evgeniya Sheremet[2], Raul D. Rodriguez[2], Aleksandar Matkovic[1*]

[1] Chair of Physics, Department Physics, Mechanics and Electrical Engineering, Montanuniversität Leoben, Franz Josef Strasse 18, 8700 Leoben, Austria.

[2] Tomsk Polytechnic University, Lenina ave. 30, 634034, Tomsk, Russia.

[3] Erich Schmid Institute of Materials Science, Austrian Academy of Sciences, Jahnstrasse 12, 8700 Leoben, Austria.

[4] Research Center for Functional Materials, National Institute for Materials Science, 1-1 Namiki, Tsukuba 305-0044, Japan.

[5] International Center for Materials Nanoarchitectonics, National Institute for Materials Science, 1-1 Namiki, Tsukuba 305-0044, Japan.

[6] Graz Centre for Electron Microscopy (ZFE), Steyrergasse 17, 8010 Graz, Austria.

[*] corresponding author's e-mails: gennadiy.murastov@unileoben.ac.at, aleksandar.matkovic@unileoben.ac.at

Supplementary Note 1

Our homemade 3D printed stage has a speed limitation as 25, 10, and 5 µm/s, high-enough position reproducibility with feasible step size of 0.5 µm. Thus, the power control over the laser beam was chosen as the primary parameter to tune the NPs deposition rate, size, and density. Based on this, we evaluate the total irradiation time normalized to the beam area, speed, and steps.

Supplementary Table 1 | Edge-specific WSe_2 NRs decorated with AgNPs within different time, speed and laser power. The table presents the original data for Fig. 3.

**Laser fluence, µJ/µm^2**	**Linear scanning speed, µm/s**	**Beam-size area irradiation time, s**	**Laser power, mW**	**NP's size, µm**	**NP's density, counts/µm**
**4.6**	10	5	~0.2	43.1 ± 20.3	**3.0 ± 1.2**
**15.3**	10	5	~0.6	34.8 ± 13.2	**5.0 ± 0.6**
**35**	25	2	~3.0	32.0 ± 10.3	**5.5 ± 2.7**
**80**	25	2	~7.8	28.6 ± 6.9	**10.7 ± 2.2**
**120**	25	2	~11.7	30.3 ± 10.6	**9.4 ± 2.0**
**300**	25	2	~29.6	31.3 ± 8.7	**11.9 ± 1.3**

.

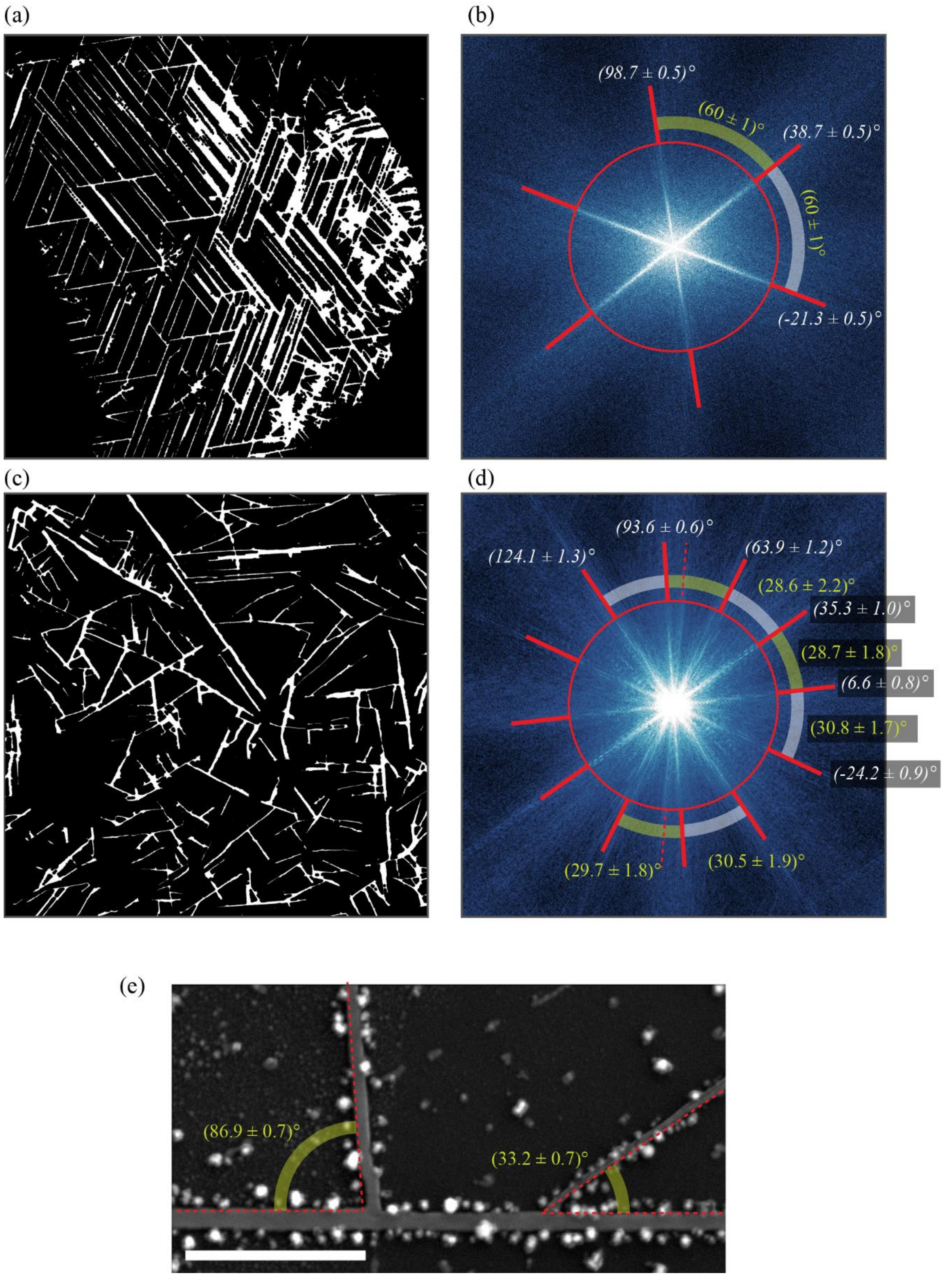

Supplementary Fig. 1 | 6P organics growth direction analysis on 2D WSe_2 fakes. a Binary map of the nanoribbons generated from a 60×60 μm^2 AFM image. **b** 2D-FFT of **a** providing the growth directions (inverse space) in white italic and the difference between the nearby registry directions in solid light green. **c** and **d** are analogous to **a** and **b** only with nanostructure self-assembly further from the equilibrium (lower deposition temperature). In case of a near-equilibrium growth of the 6P on WSe_2 (**a**, **b**) only three primary growth directions were observed, separated by 60° (**b**). These directions most likely correspond to the crystallographic zigzag direction of WSe_2, as the phenylene molecules were grown. Nanostructures assembled under non-equilibrium conditions (**c**, **d**) tend to have also the secondary set of the self-alignment directions. Consequently, the nanostructures grow with about 30° interval (**d**), and effectively alternate between predominantly armchair and zigzag directions of the nanoribbons. **e** An example of SEM micrograph (the scale bar is 1 μm) with nearly 30° and nearly 90° nanoribbon joints demonstrating that no significant difference of the predominant nanoribbon direction was observed in the NP decoration.

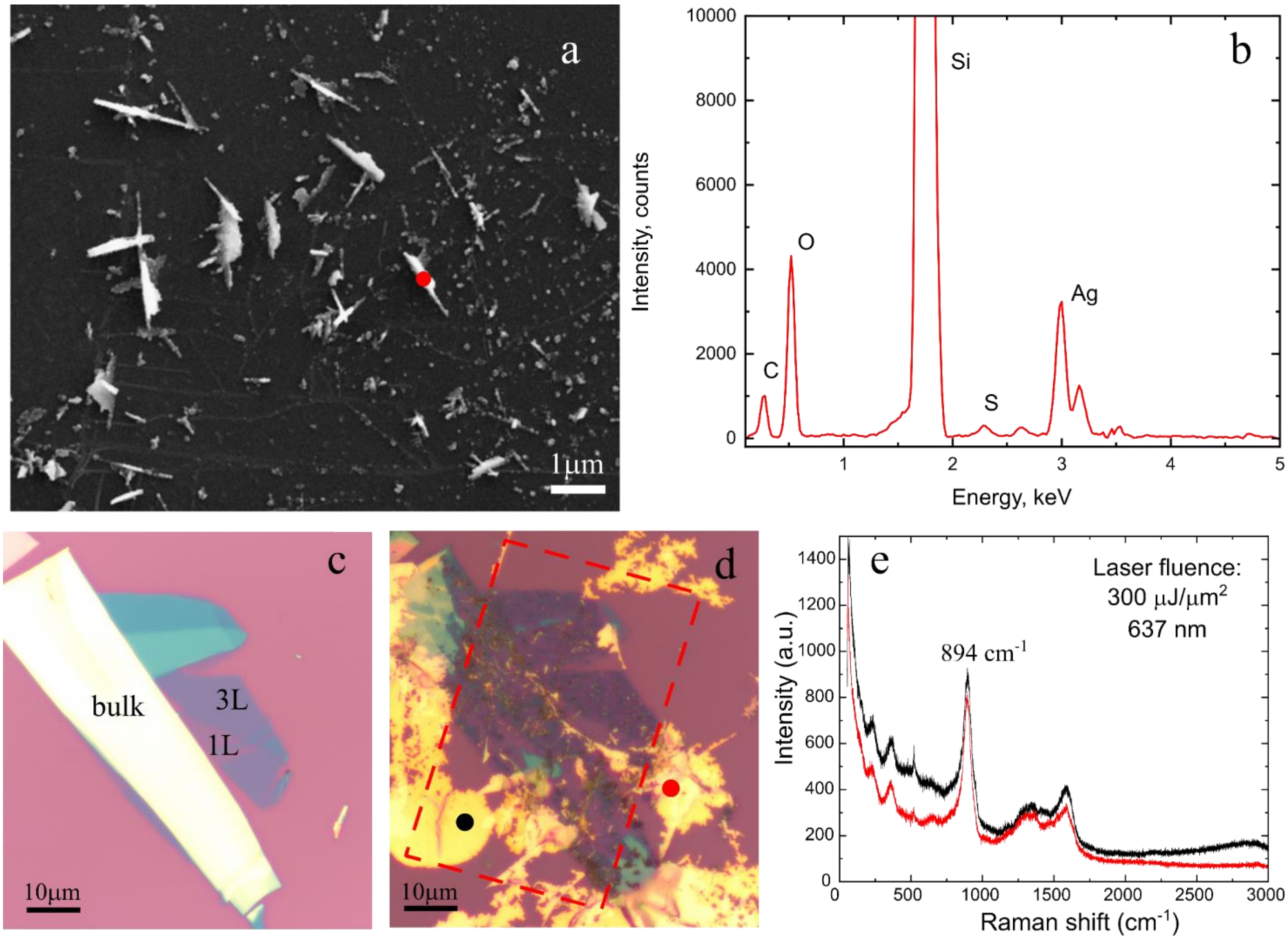

Supplementary Fig. 2 | Laser treatment of MoS_2 immersed in 1 mM $AgNO_3$. a Micrograph of a MoS_2 nanoribbon network that was exposed with laser fluence of ~20 µJ/µm^2. Instead of edge-specific growth of AgNPs (as observed on WSe_2 nanoribbons under the same conditions), in this case large elongated structures were observed. **b** EDX spectrum of these features (bright elongated structures in the SEM micrograph marked as red circle in **a** shows signature of Ag, S, and Si substrate. **c** Optical micrograph of an exfoliated MoS_2 flake with a mono and trilayer flake attached to thicker "bulk-like" crystal. **d** the same region after a 300 µJ/µm^2 Ag NP decoration attempt. Red rectangle indicates the laser scanned area. Red and black dots in **d** indicate the points where Raman spectra were taken. **e** Raman spectra of the formed dendritic features. The dominant mode at 894 cm^{-1} is associated with Ag_2MoO_4, while the peak position is associated with the β-phase[1–3].

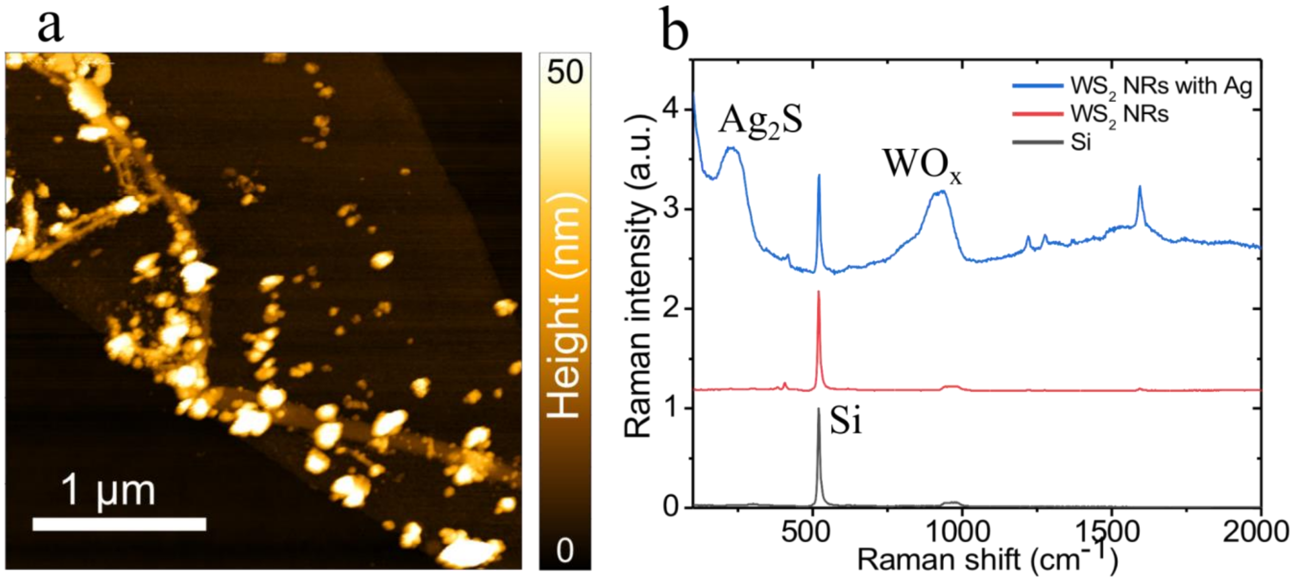

Supplementary Fig. 3 | Laser treatment of WS_2. a Topography (AFM) of a WS_2 nanoribbon network exposed with laser fluence of ~100 μJ/μm^2.**b** Comparative Raman spectra from bare Si substrate, non-treated nanoribbons, but immersed to the silver nitrate solution, and obtained tungsten oxides[4] after NP decoration attempt. In addition to WO_x broadband Raman peak from 800 to 1000 cm^{-1} there is a peak around 250 cm^{-1} attributed to the Ag_2S nanoclusters[5].

Supplementary References

1. Fabbro, M. T. *et al.* Identifying and rationalizing the morphological, structural, and optical properties of β-Ag_2MoO_4 microcrystals, and the formation process of Ag nanoparticles on their surfaces: combining experimental data and first-principles calculations. *Sci. Technol. Adv. Mater.* **16**, 065002 (2015).
2. Fabbro, M. T. *et al.* Synthesis, antifungal evaluation and optical properties of silver molybdate microcrystals in different solvents: a combined experimental and theoretical study. *Dalton Trans.* **45**, 10736–10743 (2016).
3. Wang, Z. *et al.* In situ formation of Ag_2MoO_4 in a Ag/MoO_3 buffer layer enables highly efficient inverted perovskite cell for a tandem structure. *ACS Appl. Energy Mater.* **3**, 9742–9749 (2020).
4. Kumar, P., Sarswat, P. K. & Free, M. L. Hybridized Tungsten Oxide Nanostructures for Food Quality Assessment: Fabrication and Performance Evaluation. *Sci. Rep.* **8**, 3348 (2018).
5. Sadovnikov, S. I., Rempel, A. A. & Gusev, A. I. Nanostructured silver sulfide: synthesis of various forms and their application. *Russ. Chem. Rev.* **87**, 303–327 (2018).
