# Supplementary material for: Photoinduced edge-specific nanoparticle decoration of two-dimensional tungsten diselenide nanoribbons
Source: Commun Chem. 2023 Aug 14;6:166. doi: 10.1038/s42004-023-00975-6 (PMC10425467; doi:10.1038/s42004-023-00975-6)
Supplement: Supplementary file 1 — Peer Review File [file 42004_2023_975_MOESM1_ESM.pdf]

nature portfolio

Peer Review File

Photoinduced edge-specific nanoparticle decoration of two dimensional tungsten diselenide nanoribbons

Open Access This file is licensed under a Creative Commons Attribution 4.0 International License, which permits use, sharing, adaptation, distribution and reproduction in any medium or format, as long as you give appropriate credit to the original author(s) and the source, provide a link to the Creative Commons license, and indicate if changes were made. In the cases where the authors are anonymous, such as is the case for the reports of anonymous peer reviewers, author attribution should be to 'Anonymous Referee' followed by a clear attribution to the source work. The images or other third party material in this file are included in the article's Creative Commons license, unless indicated otherwise in a credit line to the material. If material is not included in the article's Creative Commons license and your intended use is not permitted by statutory regulation or exceeds the permitted use, you will need to obtain permission directly from the copyright holder. To view a copy of this license, visit http://creativecommons.org/licenses/by/4.0/.

Reviewers' comments:

Reviewer #1 (Remarks to the Author):

The paper resides in the area of 2D materials research, specifically the use of WSe_2 nanoribbonsand their laser induced site-specific decoration with Ag nanopartricles that can be used for e.g. Surface enhanced Raman spectroscopy applications.

Here, the catalytic power of these particles is studied for an example of 4-NBT to develop into DMAB.

The results are nicely and comprehensively presented.

The results are included in a comprehensive literature collection.

I suggest to present these interesting results as they are.

Reviewer #2 (Remarks to the Author):

Reviewer’s remark
This work presents a method of decorating silver nanoparticles along WSe_2 nanoribbon edges. The array of nanoparticles was observed and their size, distribution, and photoresponse were characterized by different techniques. This approach shows high site-selectivity of nanoparticle on WSe_2 nanoribbons and the photocatalytic behavior regarding its potential applications is well demonstrated. Therefore, if the authors can well address the points below, this reviewer would recommend the acceptance of this manuscript in Communications Chemistry.

Question 1
When there is a direct laser illumination to promote a reduction of silver ion, what is the most deterministic parameter of light source that can enhance the site-selectivity and yield of nanoparticle production and what is the reason for that?

Question 2
Is this method also applicable to other two-dimensional materials, especially such as sulfur-based transition metal dichalcogenides (MoS_2 and WS_2)?

Question 3
WSe_2 has different types of edges depending on its crystal orientation, thus different types of defect sites. Authors should characterize the effect of these edge types on NP production.

Reviewer #3 (Remarks to the Author):

The paper demonstrates the formation of Ag nanoparticles on 2D WSe_2 nanoribbons via light- assisted reduction of Ag ions. The methodology has been extended to decorate the surface with Ag nanoparticles to other 2D materials such as Hexagonal Boron Nitride. The overall results are interesting. However, I believe that the results are worthy to be accepted after the following questions are addressed.

1)A reference missing on row 210.

2)The formation of Ag NPs was shown in Fig. 1 using AFM and SEM images. However, there are no any solid evidences to prove the existence of Ag NPS. Can author provide SEM mapping images and any surface analysis such as XPS to prove the formation of Ag NPS?

3)Can the author provide any time dependent decoration of Ag NPs to give supporting information into Fig. 3?

4)It is still unclear the purpose of decorating HBN with AG NPs. Can author explain this part? Will it be possible to extend the proposed methodology for other 2D materials such as MoS_2, graphene ext?

5)Author has provided only Raman spectra to demonstrate the photoactive Ag NPs. Can author provide any real time application details in this part? Such as photodetectors or HER for the bulk 4-NBT and after Ag NPs treatment?

Reviewer #4 (Remarks to the Author):

This work by Murostov et al. reports the generation and application of silver nanoparticles, specifically at the edges of WSe_2 flakes, enabled by laser-induced deposition. It seems like this work is the follow-up project of the recently published work in npj 2D materials and applications (DOI: 10.1038/s41699-022-00356-y), reporting nanoribbon field-effect transistors. Here, the main difference compared to the previous work is the usage of WSe_2 layers instead of graphene. Overall, this manuscript is well-written and clear, and I believe it is suitable for publication in Communications Chemistry with minor revisions.

1. Line 45 – Although HER is a very well-known reaction, the authors should write the full name of the reaction for potential readers who are not familiar with it.
2. Figure 1b – The right part of the figure appears to be cropped.
3. Figure 2 – Like the panels ‘a’ and ‘b’, I think it would be good to show where the Raman spectrum (black line) was measured. And in the panel ‘d’, TA(M) mode is not stated.

4. Line 322 – What is the abbreviation of 6P?
5. Can authors more suggest what is the main advantage of forming nanoparticles at the edges of flakes, rather than the topmost surfaces?

Point-by-point response
to Reviewer's questions and list of changes

Reviewer #1 (Remarks to the Author): *The paper resides in the area of 2D materials research, specifically the use of* WSe_2 *nanoribbons and their laser induced site-specific decoration with Ag nanoparticles that can be used for e.g. Surface enhanced Raman spectroscopy applications. Here, the catalytic power of these particles is studied for an example of 4-NBT to develop into DMAB. The results are nicely and comprehensively presented. The results are included in a comprehensive literature collection. I suggest to present these interesting results as they are.*

Authors' response
We are grateful for the supportive comments of the reviewer. The fact that the reviewer shares our view, strengthens our determination to pursue further research in the direction of coupled hybrid structures of metallic nanoparticles and nanostructured 2D materials.

Reviewer #2 *(Remarks to the Author): This work presents a method of decorating silver nanoparticles along WSe_2 nanoribbon edges. The array of nanoparticles was observed and their size, distribution, and photoresponse were characterized by different techniques. This approach shows high site-selectivity of nanoparticle on WSe_2 nanoribbons and the photocatalytic behavior regarding its potential applications is well demonstrated. Therefore, if the authors can well address the points below, this reviewer would recommend the acceptance of this manuscript in Communications Chemistry.*

Authors' response

We thank the reviewer for the thoughtful consideration of our manuscript. We also thank the reviewer for their careful revision of our manuscript and valuable comments that promote our research to a more logical and better-written manuscript. All the comments are addressed below, and the related changes to the manuscript are clearly specified.

Reviewer #2, Question 1: *When there is a direct laser illumination to promote a reduction of silver ion, what is the most deterministic parameter of light source that can enhance the site-selectivity and yield of nanoparticle production and what is the reason for that?*

Authors' response

The experiment offers a very large parameter space, such as laser wavelength, laser power, spot size, exposure time, $AgNO_3$ concentration, and of course the choice of the sample stack (different 2D materials, organic mask, *etc*.). Our goal was - when possible - to start from the literature reported values, and hopefully fix some of the parameters. Therefore, the laser wavelength and salt concentration were not varied in our study.

Both increase of the exposure time and increase of laser power lead to denser NP decoration. To capture both effects, we have provided the data as a function of laser fluence [*power * exposure / laser spot cross-sectional area* = $W \cdot s/cm^2$]. After the threshold of about 400 $\mu J/\mu m^2$ the density of particles per unit length of the ribbon edge is large enough that agglomeration becomes rather frequently observed. As elaborated in the main text, the Ag reduction process is driven primarily by the electrons photoexcited and emitted from the edges of the WSe_2 nanoribbons. Essentially, providing more electrons yields faster reduction, or denser edge decoration for the fixed exposure time. Similar trend of NP density to the one reported for laser fluence is expected to occur with increasing/decreasing the $AgNO_3$ concentration.

Further, edge selectivity is also promoted by the organic "cap". As discussed in the manuscript, the cap largely inhibits the basal plane decoration of the nanoribbons. However, this is the case only below a certain threshold for the laser fluence of also about 400 $\mu J/\mu m^2$; considering parahexaphenyl molecules, and fixed salt concentration. At high fluence 6P decomposition starts to occur, providing an additional source for the reduction process. This affects the NP's edge specific nucleation, as the molecular mask also becomes easier to dissolve under these conditions.

Lastly, it is important to highlight that without the electron source the process does not occur. I.e. if a clean SiO_2 surface is scanned under the conditions as provided in the study, essentially no Ag NPs were observed. This was also demonstrated on hBN nanoribbons, as a negative experiment (Fig. 4c of the main manuscript).

Changes made
To clarify the issue, and to provide further guidance for anyone looking to repeat the experiment or to adopt the procedure on another system we have made the following additions to the main text.

Changes in the main text:
Line no. 153 - 158; the following sentence was added
Direct laser illumination of the $AgNO_3$ solution does not create NPs while in presence of WSe_2 the light triggers the reduction of a silver ion to the neutral state due to release of photo-excited electrons from NRs. Ag^0 seed acts as a source for further NPs growth.

Line no. 342; the following sentence was added in the Fig. 3 caption
Irradiation with a laser fluence more than 400 μJ/μm^2 leads to NP agglomeration.

Line no. 376 - 392; the text was modified and the following sentences were added
However, at higher fluence (above 400 μJ/μm^2) 6P decomposition starts to occur providing an additional electron source for the reduction process. This yields also non-edge-specific deposition on the ribbon's basal plane as well as on the bare substrate near the ribbons.
The deposition process on hBN capped with 6P is ~5 times less efficient in comparison to WSe_2 (Fig. 4a and 4b) as the process mostly is driven by the 6P source.

Reviewer #2, Question 2: *Is this method also applicable to other two-dimensional materials, especially such as sulfur-based transition metal dichalcogenides (MoS_2 and WS_2)?*

Authors' response
This is an interesting point that we did not discuss in the original manuscript in order to keep the focus on the main results. We have performed similar experiments on MoS_2 and WS_2 nanoribbon networks, applying the same wavelength, silver nitrate solution, and the range of laser fluence. Contrary to the WSe_2, MoS_2 is easily decomposed under such conditions forming complex molybdenum oxides that incorporate also Ag, that grow as elongated or dendritic features spanning from few hundred of nanometers to few micrometers in size. These seemingly nucleate from the ribbon edges. Fig. R1a presents a SEM image of the typical structures obtained on MoS_2 nanoribbons under the decoration conditions similar to those presented in Fig. 3a of the main manuscript (decorated WSe_2 nanoribbons). An EDX spectrum from one of the elongated microstructures (marked as red dot in a) shows signature of Ag, S, and Si substrate (Fig. R1b).

Further increase of the laser fluence yields much more pronounced damage of MoS_2. To demonstrate this, we have exposed a pristine exfoliated MoS_2 flake (without organic nanostructures or etching) to high laser fluence of 300 μJ/μm^2. The results are presented in Fig. R1c-e. Large dendritic islands with similar signatures to [DOI: 10.1039/C7TC01863K] and the elongated structures in Fig. R1a have been observed. According to the Raman data an Ag_2MoO_4 (other notations are β-Ag_2MoO_4 and Ag:$AgMoO_4$) is formed and the peak position of 893-894 cm^{-1} is associated to the symmetric MoO_4 stretching (also labeled as A_{1g} mode) [DOI: 10.1021/acsaem.0c01346, DOI: 10.1039/c6dt00343e, DOI: 10.1088/1468-6996/16/6/065002].

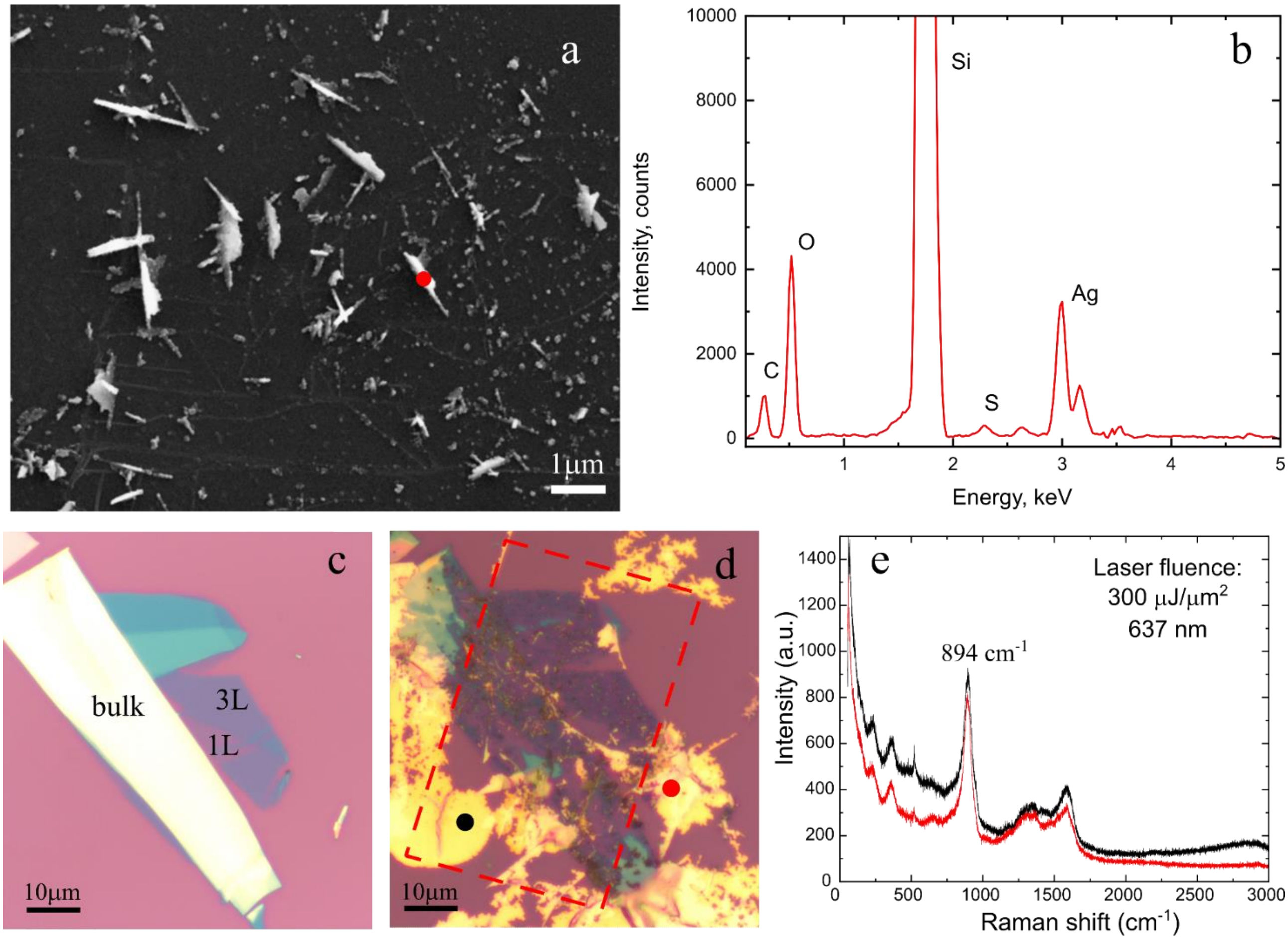

Fig. R1 Laser treatment of MoS_2 immersed in 1 mM $AgNO_3$. a Micrograph of a MoS_2 nanoribbon network that was exposed with laser fluence of ~20 μJ/μm^2. Instead of edge-specific growth of AgNPs (as observed on WSe_2 nanoribbons under the same conditions), in this case large elongated structures were observed. **b** EDX spectrum of these features (bright elongated structures in the SEM micrograph marked as red circle in **a** shows signature of Ag, S, and Si substrate. **c** Optical micrograph of an exfoliated MoS_2 flake with a mono and trilayer flake attached to thicker "bulk-like" crystal. **d** the same region after a 300 μJ/μm^2 Ag NP decoration attempt. Red rectangle indicates the laser scanned area. Red and black dots in **d** indicate the points where Raman spectra were taken. **e** Raman spectra of the formed dendritic features. The dominant mode at 894 cm^{-1} is associated with Ag_2MoO_4, while the peak position is associated with the β-phase.

Moreover, at higher laser fluence of ~300 μJ/μm^2, bulk-like flakes (over 20 layers) were almost completely removed in the scanned region, leaving only oxidized molybdenum-silver bulk signature as demonstrated in Fig. R1d. No degradation of MoS_2 was observed on the flakes (or ribbons) that were submerged into $AgNO_3$ solution, but were far from the laser illumination area.

Further, we have obtained preliminary data on WS_2 decorated nanoribbons that match the observations made on MoS_2. Atomic force microscopy (AFM) and Raman spectra from clean substrate, nanoribbons, and laser treated regions are presented in Fig. R2. We observed new oxides mode formation as a WO_x peak in a range of 800 to 1000 cm^{-1} [DOI:10.1038/s41598-018-21605-5]. This result is similar to the MoS_2 oxidation which confirms the light-induced decomposition of 2D sulfur-based material. Moreover, another band around 250 cm^{-1} is presented in Raman spectra that could be assigned to the sulfides cluster's growth during the harsh laser scanning of WS_2 [DOI: 10.1070/RCR4803].

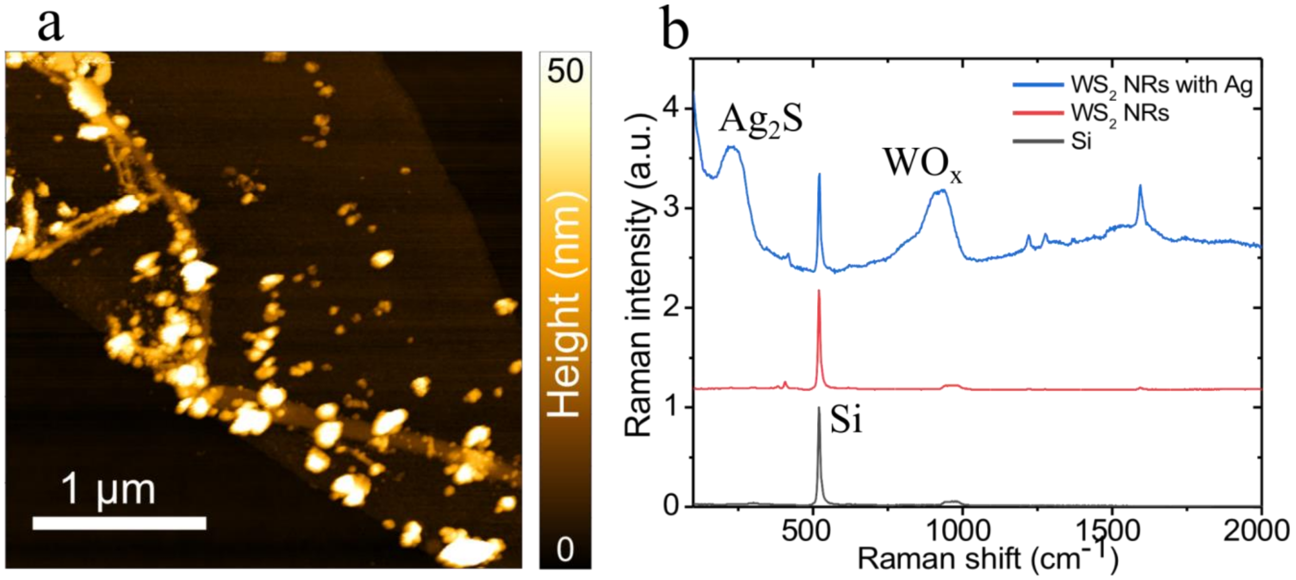

Fig. R2 Laser treatment of WS_2. a Topography (AFM) of a WS_2 nanoribbon network exposed with laser fluence of ~100 µJ/µm^2.**b** Comparative Raman spectra from bare Si substrate, non-treated nanoribbons, but immersed to the silver nitrate solution, and obtained tungsten oxides after NP decoration attempt. In addition to WO_x broadeband Raman peak from 800 to 1000 cm^{-1} there is a peak around 250 cm^{-1} attributed to the Ag_2S nanoclusters.

Finally, we were impressed by $PtSe_2$ stability within given silver nitrate solution concentration, laser fluence range, and laser wavelength. NRs networks made from $PtSe_2$ demonstrate almost no NP decoration, and at the same time we did not observe any damage to the nanoribbons. We attribute this to the different light-excitation nature of $PtSe_2$ meaning the source and irradiation parameters need to be tuned to obtain the NPs growth. Such extension, while surely interesting to explore, is beyond the scope of this study.

Changes made

To address this issue and to give a short point to the readers about our findings on MoS_2 and WS_2 two supplementary figures were introduced. Also, we briefly mentioned complex oxide formation, contrary to WSe_2 decoration explained in the main text.
Our findings of $PtSe_2$ decoration and its stability are addressed in the main text.

Changes in the main text

Line no. 395 - 400; the following sentence was added

Opposite results were obtained for MoS_2 and WS_2 NRs. In these cases, and within the explored parameter space, the sulfide based 2D materials were easily oxidized forming the Ag_2MoO_4 dendrites and WO_x, Ag_2S structure as shown in Supplementary Fig.-s 2 and 3.

Line no. 408 - 410; the following changes was implemented

Almost zero AgNPs were observed on the cleaned hBN NRs, even with a higher laser fluence ~347 µJ/µm^2 applied. *Interestingly, the same finding was obtained on $PtSe_2$ NRs likely due to lower light-matter interaction.*

Addition in the supporting information

The Fig. R1 was added in the SI file as a Supplementary Fig. 2.
The Fig. R2 was added in the SI file as a Supplementary Fig. 3.

Reviewer #2, Question 3: *WSe_2 has different types of edges depending on its crystal orientation, thus different types of defect sites. Authors should characterize the effect of these edge types on NP production.*

Authors' response

We agree with the reviewer, and we have expected similar results ourselves in the process of planning this study. However, we have not observed a clear difference in NP deposition between the different predominant nanoribbon edge directions. We have also carried out a cross-check experiment with SF_6 etching (instead of O_2 etched ribbons that are presented in the manuscript) and have obtained the NP decoration within the sample-to-sample variations of the size and density observed for the O_2 etched ribbons. For the sake of clarity, we focus only on O_2 etching in the manuscript.

When organic nanostructures self-assemble on 2D materials (2D van der Waals surfaces), they tend to follow a particular epitaxial (self-aligning) pattern. This defines a predominant nanoribbon direction. Commonly using phenylene (armchair-like) or acene (zigzag-like) backbone of the organic molecules leads to the nanoribbon growth in zigzag or armchair directions, respectively. We have demonstrated this in our previous study [DOI: 10.1038/s41699-022-00356-y] (*e.g.* see Fig. 3 in the referenced work).

However, we do not expect that our method produces atomically sharp ribbon edges, and the properties (as reactivity or edge defect density) of the edges are not sufficiently pronounced to induce a detectable difference in the NP growth. With better control of the edge type, edge termination species, and tuned parameters of the growth we expect it would be possible to also impact the NP decoration to some extent.

Changes made

To address the impact of the predominant edge direction on the NP assembly, we have provided a short discussion in the main text, and have added a supporting information as Supplementary Fig. 1. The Fig. R3 provides the information on the self-assembly pattern, *i.e.* on the predominant growth directions of the nanostructures, and then focuses on the near 90° joints between the ribbons, as these would have alternation between zigzag and armchair predominant crystallographic growth direction. No significant difference in the NP assembly was observed in such cases.

Changes in the main text

Line no. 284 - 289; the following sentence was added

Also, NPs growth was found to be very similar on both predominantly zigzag and armchair edge-types. This can be seen at ~90° joints between the NRs from Fig. 3b and c (more details in Supplementary Fig. 1).

Addition in the supporting information

The following Fig. R3 and its caption were added into the Supporting information file.

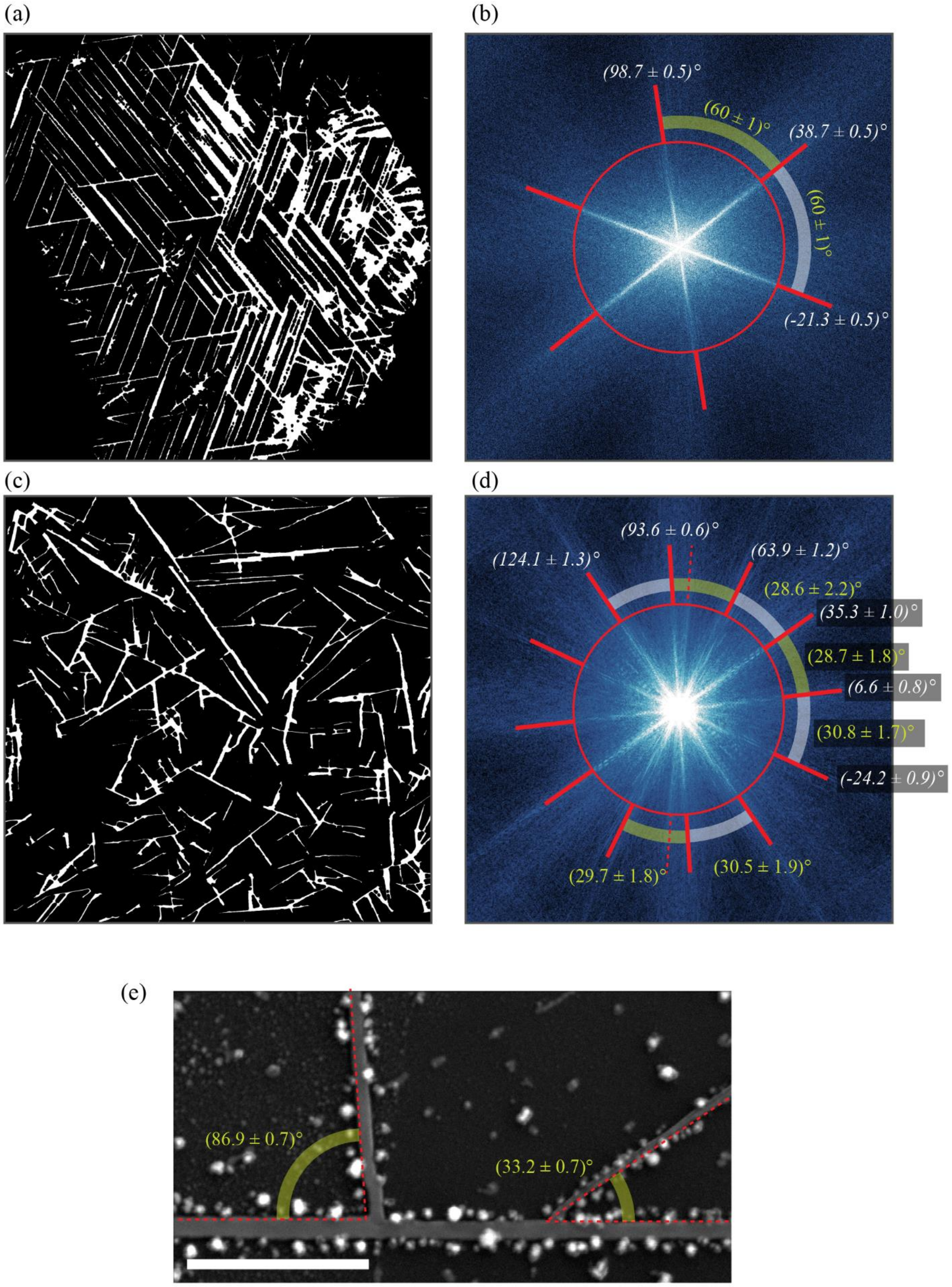

Fig. R3 6P organics growth direction analysis on 2D WSe_2 fakes. a Binary map of the nanoribbons generated from a 60×60 μm^2 AFM image. **b** 2D-FFT of **a** providing the growth directions (inverse space) in white italic and the difference between the nearby registry directions in solid light green. **c** and **d** are analogous to **a** and **b** only with nanostructure self-assembly further from the equilibrium (lower deposition temperature). In case of a near-equilibrium growth of the 6P on WSe_2 (**a**, **b**) only three primary growth directions were observed, separated by 60° (**b**). These directions most likely correspond to the crystallographic zigzag direction of WSe_2, as the phenylene molecules were grown. Nanostructures assembled under non-equilibrium conditions (**c**, **d**) tend to have also the secondary set of the self-alignment directions. Consequently, the nanostructures grow with about 30° interval (**d**), and effectively alternate between predominantly armchair and zigzag directions of the nanoribbons. **e** An example SEM micrograph (please add scale here) with nearly 30° and nearly 90° nanoribbon joints demonstrating that no significant difference of the predominant nanoribbon direction was observed in the NP decoration.

***Reviewer #3** (Remarks to the Author): The paper demonstrates the formation of Ag nanoparticles on 2D WSe_2 nanoribbons via light- assisted reduction of Ag ions. The methodology has been extended to decorate the surface with Ag nanoparticles to other 2D materials such as Hexagonal Boron Nitride. The overall results are interesting. However, I believe that the results are worthy to be accepted after the following questions are addressed.*

Authors' response

We thank the reviewer for the positive feedback and the critical view of our work. We have tried to meet most of the requests by the referee, however some points would go much beyond the scope of our study. We hope that all points are clarified.

***Reviewer #3, Question 1:** A reference missing on row 210.*

Authors' response

The references supporting the claims in the sentence on lines 207-210 are the ones that also support the claims in the following sentence (lines 210-213). We see how the statement was not clear in the original manuscript.

Changes made

To clarify the supporting references to the part of the sentence ending on line 210 (of the original manuscript) we have merged it with the following sentence.

Changes in the main text

Line no. 217 - 219; "The overtone of the LA phonon branch at the M point of the Brillouin zone can be observed *in the Raman spectra. It is commonly noted as 2LA(M) alongside* the prominent B_{2g}^1 peak which is associated with an A-symmetry first-order mode corresponding to an interlayer vibration[18,45-48]"

***Reviewer #3, Question 2:** The formation of Ag NPs was shown in Fig. 1 using AFM and SEM images. However, there are no any solid evidences to prove the existence of Ag NPS. Can author provide SEM mapping images and any surface analysis such as XPS to prove the formation of Ag NPs?*

Authors' response

We are very grateful to the reviewer for such a crucial point on the confirmation of silver nanoparticles. Within our original consortia, we did lack a capability to confirm directly the presence of Ag in the nanoparticles observed by AFM and SEM. Indications from the SEM contrast, SERS signal, and the capability of photocatalysis all indirectly supported that the particles were indeed Ag.

To address this issue directly, we have outreached to our collaborators, and have performed Energy Dispersive X-ray (EDX) spectroscopy and EDX spectral mapping of both sample regions presented in the original manuscript as well as freshly prepared samples. The EDX maps clearly correlate Ag $L\alpha_1$ peak with the previously observed nanoparticles, in contrast to *e.g.* Se L peaks that are confined to the ribbons. We agree with the referee that XPS would give us more insights into the binding and oxidation of Ag, however, to achieve the needed lateral resolution we would need to request a large-scale facility experiment (access to X-ray beam lines), that if successful would likely postpone the

study by at least 6 to 12 months. Please mind that our nanoribbon samples are fabricated from exfoliated flakes, so the total sample area of the nanoribbon networks decorated with Ag nanoparticles are in the order of few 100 μm^2 and using laboratory-scale XPS instruments would probe much larger area, therefore losing the nanoscale mapping and potentially also capturing the signal outside of our region of interest. Therefore, we have chosen to support our findings with EDX.

Changes made

To directly prove that the presented nanoparticles in our study are composed of Ag, and that Ag is not deposited also elsewhere on the sample's surface (in the vicinity of the ribbons or on the ribbons) we have expanded Fig. 2 and the corresponding paragraph in the main text

Changes in the main text

Line no. 201; Panels **e**, **f**, and **g** were added to the Fig. 2

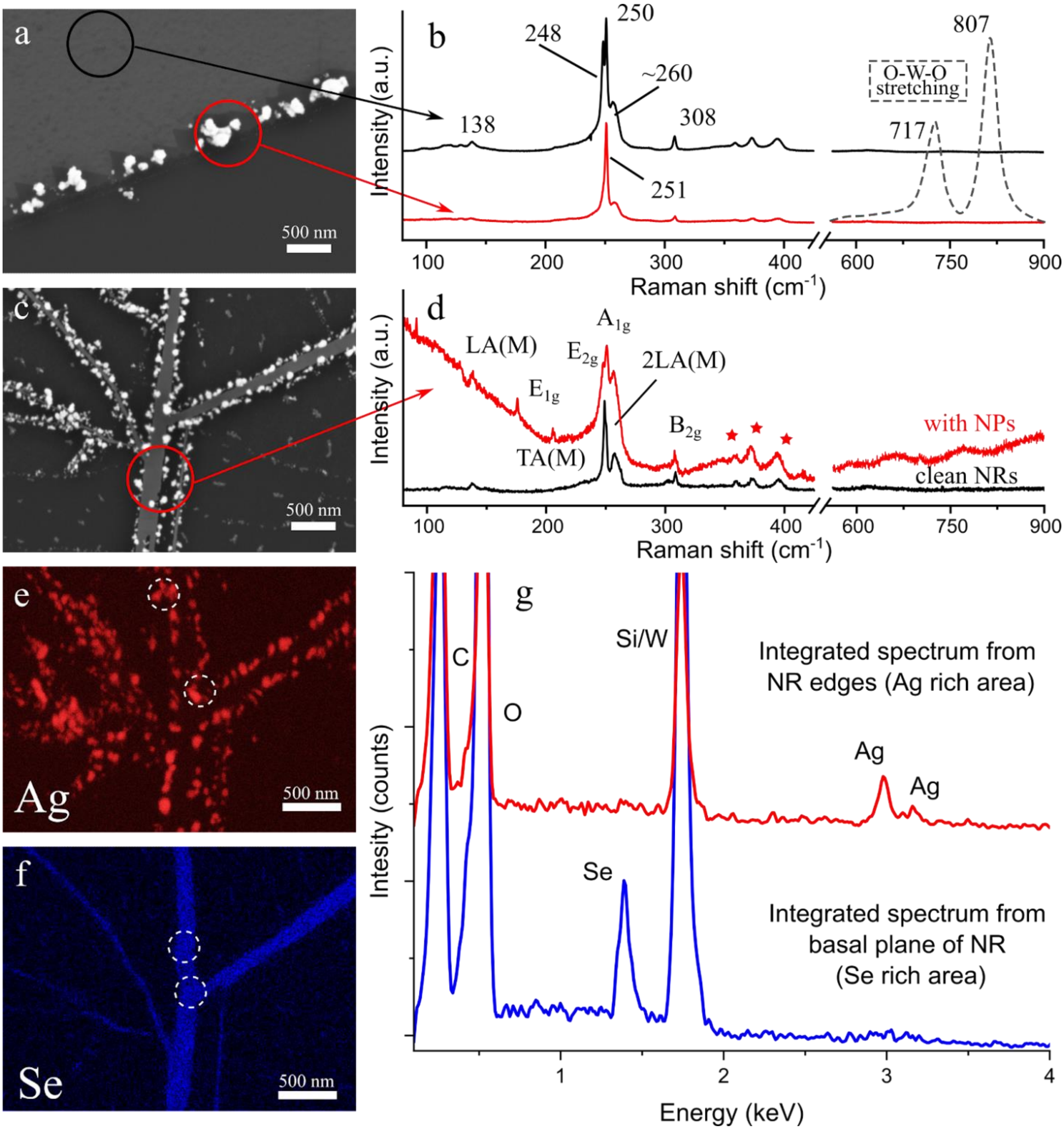

Line no. 208 – 210; Extended the caption to the Fig. 2. ***e*** *EDX elemental maps performed for silver* ***e*** *and selenium* ***f*** *at the same area as on panel* ***c****.* ***g*** *Corresponding energy dispersive X-ray spectra to the NR edges (Ag rich area) and NR basal plane (Se rich area).*

Line no. 254 – 264; A brief explanation of the EDX data was added.

The same WSe_2 NR network as for Fig. 3c was analyzed by energy dispersive X-ray (EDX) spectrometry. The presence of silver is confirmed by tracking the Ag L peak during the map measurements. To obtain a better signal to noise ratio, spectral map points from the areas labeled with dashed circles in Fig. 2e were merged and are presented as an integrated spectrum in Fig. 2g. The same is done for Se L peak from the basal plane of the NR given in Fig. 2f.

Changes in the Methods section

Line no. 576 – 582; EDX equipment was added.

Zeiss Sigma 300 VP coupled with EDS Detector Oxford SDD 80 was used to obtain the elemental map from the decorated WSe_2 nanoribbons covered within 5 – 10 nm carbon coating to minimize surface changing and image drifting during the long-term measurements.

Reviewer #3, Question 3: *Can the author provide any time dependent decoration of Ag NPs to give supporting information into Fig. 3?*

Authors' response

Our homemade 3D printed stage has a speed limitation as 25, 10, and 5 µm/s, high-enough position reproducibility with feasible step size of 0.5 µm. Thus, the power control over the laser beam was chosen as the primary parameter to tune the NPs deposition rate, size, and density. Based on this, we evaluate the total irradiation time normalized to the beam area, speed, and steps. The table below presents the original data from Fig. 3.

Laser fluence, µJ/µm^2	Linear scanning speed, µm/s	Beam-size area irradiation time, s	Laser power, mW	NP's size, µm	NP's density, counts/µm
**4.6**	10	5	~0.2	43.1 ± 20.3	**3.0 ± 1.2**
**15.3**	10	5	~0.6	34.8 ± 13.2	**5.0 ± 0.6**
**35**	25	2	~3.0	32.0 ± 10.3	**5.5 ± 2.7**
**80**	25	2	~7.8	28.6 ± 6.9	**10.7 ± 2.2**
**120**	25	2	~11.7	30.3 ± 10.6	**9.4 ± 2.0**
**300**	25	2	~29.6	31.3 ± 8.7	**11.9 ± 1.3**

Changes made

To show the influence of irradiation time for NPs growth, the dataset from Fig. 3 was extended to the full list (including, time, laser power, scan speed) and assembled in one Supporting information table.

Changes in the main text

Line no. 277 – 280; The following sentence was added:

The results *presented in Fig. 3 given as a function of laser fluence, while all varying parameters are included in the Supplementary Table S1*.

Addition in the supporting information

The full set table was added in the SI file as a Supplementary Table S1.

Reviewer #3, Question 4: *It is still unclear the purpose of decorating hBN with Ag NPs. Can author explain this part? Will it be possible to extend the proposed methodology for other 2D materials such as MoS_2, graphene ext?*

Authors' response

We agree with the referee that decorating hBN (an insulator) does not have as many potential applications as 2D semiconductors (especially ambipolar WSe_2). However, the experiments summarized in Fig. 4 and its corresponding section give many insights into the electron source for the reduction process.

To confirm that the light-induced reduction is governing the process and that the electron source is WSe_2, we have conducted negative NP decoration experiments on 2D hBN nanoribbon networks. Due to the dielectric nature of hBN it cannot be excited by visible light to provide an electron for silver seeding. On the other hand, the nanoribbon preparation method is almost identical to WSe_2 ribbons, and we assume that the major difference is in the removal of photoexcited electrons from the ribbons.

This result is demonstrated on the Fig. 4c in the main text. However, we still see the NP growth on the hBN nanoribbons capped with an organic layer of 6P. This gave us an opportunity to estimate the contribution in the NP seeding by the organic cap. We expect a similar amount of the seeding contribution to be initiated also with other 2D materials, considering the same NP decoration parameters. Contrary to the hBN ribbons with the organic caps, clean hBN flakes or nanoribbons do not promote NP particle growth at all. Simply, without the photoexcited electron source the silver ions are not reduced, and have a strong repulsive force that prohibits the formation of NPs.

Other TMDCs:

We have tested several other 2D materials, however, to have a concise and thoroughly carried out study we have decided to focus our work on WSe_2 nanoribbons. We have obtained the most controllable and repeatable NP growth on WSe_2 within the explored parameter range (laser wavelength, fluence range, salt concentration). Below we summarize our efforts to decorate other 2D materials besides WSe_2 and hBN. Please also refer to the ***Reviewer #2, Question 2*** and our reply, as it covers mostly the extension to sulfide based TMDCs (MoS_2, WS_2) as well as $PtSe_2$.

According to our preliminary results, MoS_2 is highly oxidized and forms Ag_2MoO_4 under the same laser treatment as WSe_2. That leading to dendrites structure growths upon the destructed layers. Nevertheless, we believe the methodology itself could be extended for MoS_2 NP decoration as well. However, it would require a significantly different parameter set, considering laser power, wavelength, and solution (salt concentration) ranges other than those investigated in our study.

As it was shown in our previous study [DOI: 10.1038/s41699-022-00356-y] graphene NRs are possible to decorate with Ag NPs within our parameter range. Similar to WSe_2, graphene NRs show the edge-specific growth of NPs. Interestingly, we have observed that under the same conditions the organic cap is less stable on graphene than on WSe_2. We could induce a significant removal of the organic cap by laser scanning in the solution. We hypothesize that the registry between graphene and 6P is not as well matched as in the case of WSe_2, and also that 6P exposes a different top crystallographic plane on graphene than on WSe_2. Further, we could consider that graphene could heat more under the illumination (higher number of free carriers) and that would enhance the molecule desorption process too. This mainly affects the edge-specific selectivity and the repeatability of the process, as the removed molecules can introduce nucleation centers on the free

substrate surface surrounding the NRs. The summarized results on graphene NR treated with laser are demonstrated on the Fig. R4 and in the Supplementary Fig. 2.

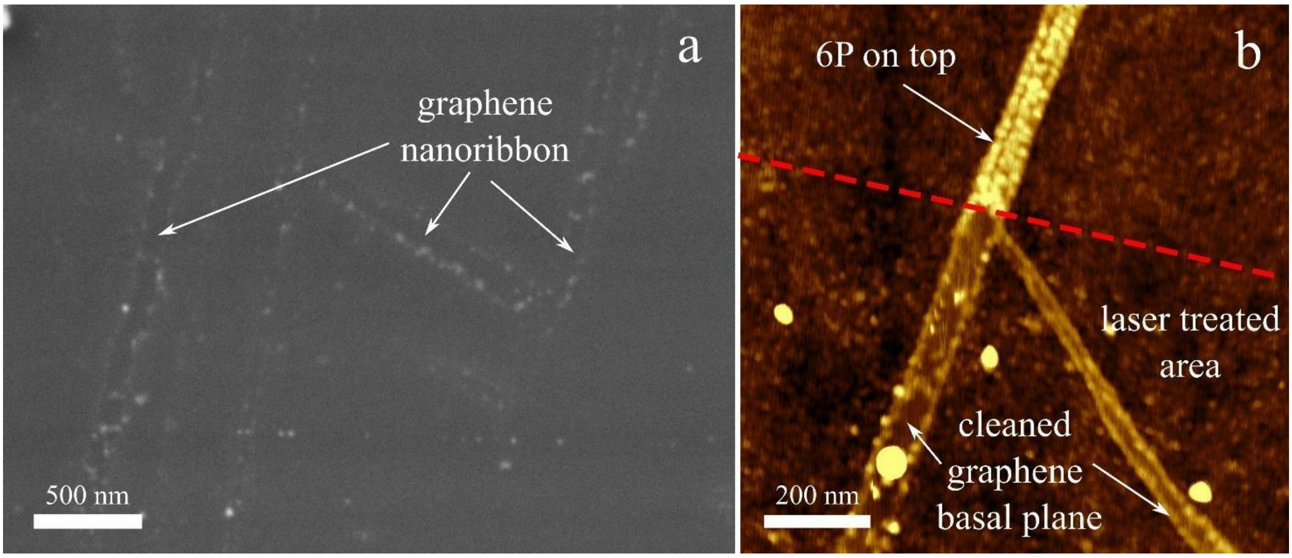

Fig. R4 SEM and AFM images of graphene nanoribbons decorated with AgNPs. a Edge-specific decoration of graphene nanoribbon network irradiated with 637 nm laser diode, full-area was scanned in the silver nitrate solution. **b** Ablation of 6P organic caps during the decoration process on graphene. Mind that the significant organic molecule desorption affects the edge-specific nucleation, as this process occurs in-line with metallic NPs edge decoration.

Changes made

The choice of hBN as a 2D insulator that cannot provide an electron for the reduction process is explained more directly.

Considering the discussion and changes made on the ***Reviewer #2, Question 2***, ***Reviewer #2, Question 3*****,** and in this section, we want to emphasize the tunability of proposed methodology and applicability to the different 2D material NRs decoration. Also, the method could be extended (repurposed) and used for example as organic residue cleaning or precise oxide formation.

Changes in the main text

Line no. 353; To investigate the impact of 6P on the proposed laser-assisted NP growth, we have performed a *negative experiment* on hexagonal boron nitride NRs with and without 6P caps (Fig. 4).

Line no. 367 – 368; The following sentence was moved to the end of paragraph to improve the text readability and emphasize the negative experiments with hBN decoration.

Therefore, we could test the influence of the 6P caps in NP seeding.

__Reviewer #3, Question 5:__ Author has provided only Raman spectra to demonstrate the photoactive Ag NPs. Can author provide any real time application details in this part? Such as photodetectors or HER for the bulk 4-NBT and after Ag NPs treatment?

Authors' response

We highly appreciate this question, as this is indeed our planned continuation to develop the topic further and also our original intention for the application of this hybrid system.

Therefore, here we want to share our preliminary results on the light-response of a working NP decorated NR device (mind without enough device statistic data). Fig. R5 demonstrates the WSe_2 FET device decorated with AgNPs along the nanoribbon network. The IV curves from a pristine device show small increase in the current of the p-branch while operating under constant illumination of a white LED source, and in comparison, to the transfer curves measured in the dark. After AgNPs decoration the prominent increase in the response of the n-branch was observed coming from metallic shared electrons (black curve on Fig. R5a). Upon white light illumination the electron branch exhibits about one order of magnitude enhanced conductivity, and the hole branch exhibits only a minor increase (50%) - please see solid orange and black curves in Fig. R5a.

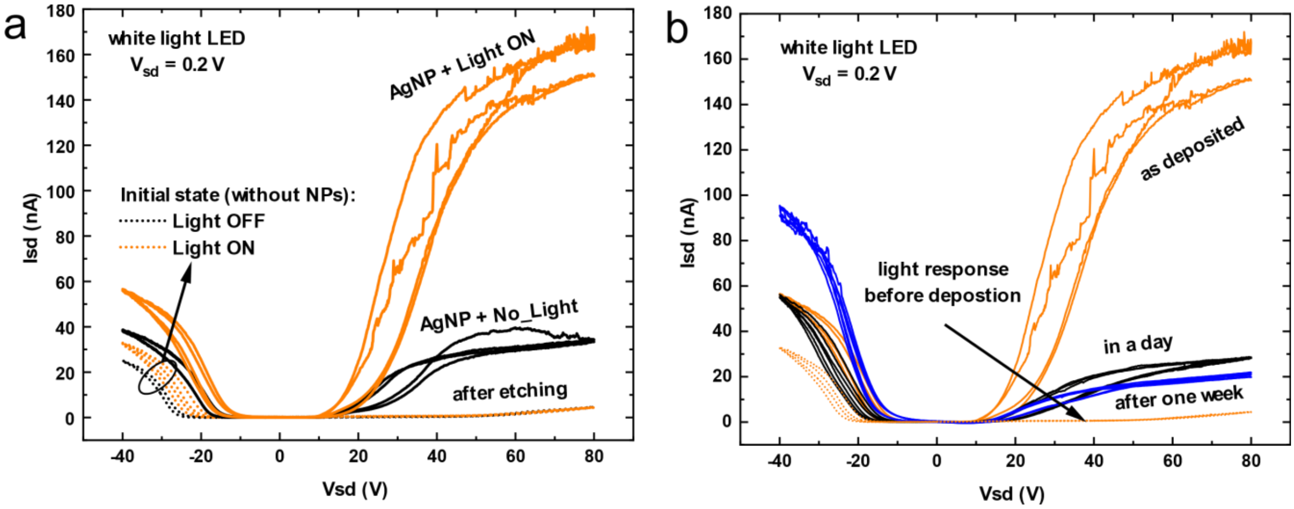

Fig. R5. Electrical performance of a WSe_2 FET assisted with white light irradiation. **a** Photo-response enhancement after decoration with AgNPs. The electronic branch is highly doped by the hot plasmon electron. The I_{sd} current is ~100 times higher with AgNPs decoration and light. **b** Degradation of electronic conductivity of the device over the time.

We also notice the fast NPs degradation by time-tracking the evaluation of electrical response within a week while our device was stored in the ambient conditions. Fig. R5b demonstrates the conductivity change for both p- and n-type of WSe_2 FET during the time. As fresh Ag NPs were deposited the electronic branch is highly doped, while this effect is diminished through the time due to the silver oxidation in air.

Here we briefly discussed our preliminary results obtained from on WSe_2 FET device. Overall results are interesting and have a potential for further investigations as a separate topic. However, we do not see fitting that these preliminary results on one device are suitable to be included in this publication. Thus, we decide do not include any FET's data in our current manuscript (nor Supplementary information file). We currently work on understanding the functionality of these hybrid FETs. For this we need to carry out a systematic study with sufficient statistics, probing sensitivity, enhancement, and wavelength response of decorated devices. These will be reported in a separate (follow-up) study.

***Reviewer #4** (Remarks to the Author): This work by Murastov et al. reports the generation and application of silver nanoparticles, specifically at the edges of WSe_2 flakes, enabled by laser-induced deposition. It seems like this work is the follow-up project of the recently published work in npj 2D materials and applications (DOI: 10.1038/s41699-022-00356-y), reporting nanoribbon field-effect transistors. Here, the main difference compared to the previous work is the usage of WSe_2 layers instead of graphene. Overall, this manuscript is well-written and clear, and I believe it is suitable for publication in Communications Chemistry with minor revisions.*

Authors' response
We thank the reviewer for their positive feedback on our current and previous work and for providing the critical comments to improve the manuscript. We addressed all reviewer's questions point by point in the following section. Please, mind the cross-linked answers to the similar questions from other reviewers.

***Reviewer #4, Question 1:** Line 45 – Although HER is a very well-known reaction, the authors should write the full name of the reaction for potential readers who are not familiar with it.*

Authors' response
We thank the reviewer for pointing this out.

Changes made
Abbreviation "HER" is now defined.

Changes in the main text
Line no. 46; the following text was added: "...from *hydrogen evolution reaction (HER)* to…".

***Reviewer #4, Question 2:** Figure 1b – The right part of the figure appears to be cropped.*

Authors' response
We thank the reviewer for his/her comment. The problem was with the wrong layer order between Fig. 1b and Fig. 1c in the graphics editing software, which then causes the white background overlapping with the text. Now all labels are visible and readable.

Changes made
Fig. 1 was reassembled with correct labels.

Changes in the main text
Line no. 139; Fig. 1 was replaced by a new one without the cropped text issue.

***Reviewer #4, Question 3:** Figure 2 – Like the panels 'a' and 'b', I think it would be good to show where the Raman spectrum (black line) was measured. And in the panel 'd', TA(M) mode is not stated.*

Authors' response
To be sure that we are not overlapping with the Raman signal from decorated nanoribbons presented on the Fig. 2c, we perform the measurement outside the scanned nanoribbons network what is also

out of the presented image. We also prefer to keep image on the panel **c** within a high magnification to highlight the presence of NPs. Thus, the point out both regions in one image is not possible, but to improve the readability of the text and figure we introduce labels on the spectra presented on the panel **d**. We hope that now it provides the clear picture from both decorated and non-treated areas assigned to the Raman spectra.

Changes made

Fig. 2 was revised according to the reviewer's comment. A TA(M) peak is assigned and the Raman spectra (both red and black lines) are properly labeled on Fig. 2.

Changes in the main text

Line no. 201; Fig. 2 was renewed. The sub-panels that have raised the issue here are kept in the updated figure with the described changes. Please, refer, also to the changes given in ***Reviewer #3, Question 2*****.**

Reviewer #4, Question 4: *Line 322 – What is the abbreviation of 6P?*

Authors' response:

"**Para-hexaphenyl**" - 6P or p-6P ($C_{36}H_{26}$) - consists of six phenyl rings connected by single sp^2 carbon- carbon bonds forming a rod-like structure with the dimensions 2.66, 0.49 and 0.33 nm (length, wide and height). The other naming of this organic structure includes: p-Sexiphenyl, p-Hexaphenyl, 1,1':4',1'':4'',1''':4''',1'''':4'''',1'''''-Sexiphenyl, *etc.*

Changes made

Additional explanation of organics structure is introduced in the Methods section.

Changes in the main text

Line no. 119: We give both names in the following way: '…para-hexaphenyl $C_{36}H_{26}$ (6P*, p-6P*) grown…'.

Changes in the Methods section

Line no. 532 – 535; Briefly, hot wall epitaxy was used to deposit the organic mask containing *6P (p-6P or $C_{36}H_{26}$ consists of six phenyl rings connected by single sp^2 carbon- carbon bonds forming a rod-like conjugated molecule)* on top of mechanically exfoliated 2D flakes (WSe_2, *MoS_2, graphene, and* hBN) followed by reactive oxygen ion etching.

Reviewer #4, Question 5: *Can authors more suggest what is the main advantage of forming nanoparticles at the edges of flakes, rather than the topmost surfaces?*

Authors' response

There are several advantages of edge-specific decoration in opposition to the topmost surface decoration:

- ✔ First, and foremost, the basal plane of the nanoribbon is protected by 6P and it remains unaffected by the decoration process, *i.e.* the intrinsic 2D material is not decoupled or damaged by laser. We expect that this will yield much better electronic properties of the WSe_2 nanoribbons than of the basal plane decorated thin films. Decoration of the basal plane can

lead to damage of the 2D semiconductor crystal structure and therefore an increased scattering rate of the free carriers. Consequently, the electronic properties of the 2D semiconductor could be significantly degraded making potential electronic excitation or readout impossible.

- ✔ Electronic coupling via the edges is expected to be more efficient than over the basal plane, as also conductivity of most 2D materials is much higher in than out of plane. Therefore, nanoribbons with edge nanoparticle decoration maximize the interaction via the edges due to the high edge-to-surface ratio. We expect that these hybrid systems can be used in sensing, controllable catalysis, plasmonics, *etc*.

- ✔ Once attached to the WSe_2 NR edges, Ag NPs are stable and can withstand multiple rinsing, or submersion for further chemical reactions. Such stable anchoring without damaging the 2D material's basal plane is more robust than top surface NPs sedimentation. Consequently, edge-specific decorated structures could have much broader application scope.

We also refer to our preliminary result on WSe_2 decorated FETs ***(Reviewer #3, Question 5)*** to show the potential application and advantage of edge-decoration in electronics, sensing, plasmonic and optoelectonic devices.

Changes made
We elaborate on more advantages promoted by the edge-specific decoration instead of the top NPs coating.

Changes in the main text

Line no. 503 – 514; The following sentences were added to highlight the most crucial point about edge-specific decoration:

We expect that this will yield much better electronic properties of the WSe_2 NRs than of the basal plane decorated thin films. Decoration of the basal plane can lead to damage of the 2D semiconductor crystal structure and therefore an increased scattering rate of the free carriers. Consequently, the electronic properties of the 2D semiconductor could be significantly degraded making potential electronic excitation or readout impossible.

Line no. 523; The point of the ultra-sensitive device: '…to develop the *ultra-sensitive* devices based on…'.

REVIEWERS' COMMENTS:

Reviewer #2 (Remarks to the Author):

After the revision, I think that this work now have all the ingredients to be published in Communications Chemistry and I am confident that the readership will enjoy this work.

Reviewer #3 (Remarks to the Author):

The authors have addressed all concerns from the reviewer. It is a good shape for the publication in communications chemistry.

Point-by-point response to Reviewer's questions and list of changes (Round 2)

Reviewer #2 (Remarks to the Author): After the revision, I think that this work now have all the ingredients to be published in Communications Chemistry and I am confident that the readership will enjoy this work.

Authors' response We are grateful for the positive feedback on our corrections.

Reviewer #3 (Remarks to the Author): The authors have addressed all concerns from the reviewer. It is a good shape for the publication in communications chemistry.

Authors' response We thank the reviewer for the collaborative work leading to the improvement of our manuscript.
